# Implications of Seed Vault Storage Strategies for Conservation of Seed Bacterial Microbiomes

**DOI:** 10.3389/fmicb.2021.784796

**Published:** 2021-12-03

**Authors:** Ankush Chandel, Ross Mann, Jatinder Kaur, Sally Norton, Jacqueline Edwards, German Spangenberg, Timothy Sawbridge

**Affiliations:** ^1^Agriculture Victoria Research, AgriBio, Centre for AgriBioscience, Bundoora, VIC, Australia; ^2^School of Applied Systems Biology, La Trobe University, Bundoora, VIC, Australia; ^3^Agriculture Victoria Research, Australian Grains Genebank, Horsham, VIC, Australia

**Keywords:** seed vault, storage strategies, seed bacterial microbiomes, conservation, culturability

## Abstract

Global seed vaults are important, as they conserve plant genetic resources for future breeding to improve crop yield and quality and to overcome biotic and abiotic stresses. However, little is known about the impact of standard storage procedures, such as seed drying and cold storage on the seed bacterial community, and the ability to recover seed-associated bacteria after storage. In this study, soybean [*Glycine max* (L.) Merr.] seeds were analyzed to characterize changes in the bacterial community composition and culturability under varying storage conditions. The *G. max* bacterial microbiome was analyzed from undried seed, dried seed, and seed stored for 0, 3, 6, and 14months. Storage temperatures consisted of −20°C, 4°C, and room temperature (RT), with −20°C being commonly used in seed storage vaults globally. The seed microbiome of *G. max* was dominated by *Gammaproteobacteria* under all conditions. Undried seed was dominated by *Pantoea* (33.9%) and *Pseudomonas* (51.1%); however, following drying, the abundance of *Pseudomonas* declined significantly (0.9%), *Pantoea* increased significantly (73.6%), and four genera previously identified including *Pajaroellobacter*, *Nesterenkonia*, env.OPS_17, and *Acidibacter* were undetectable. Subsequent storage at RT, 4, or −20°C maintained high-abundance Genera at the majority of time points, although RT caused greater fluctuations in abundances. For many of the low-abundance Genera, storage at −20°C resulted in their gradual disappearance, whereas storage at 4°C or RT resulted in their more rapid disappearance. The changes in seed bacterial composition were reflected by cultured bacterial taxa obtained from the stored *G. max* seed. The main taxa were largely culturable and had similar relative abundance, while many, but not all, of the low-abundance taxa were also culturable. Overall, these results indicate that the initial seed drying affects the seed bacterial composition, suggesting that microbial isolation prior to seed drying is recommended to conserve these microbes. The standard seed storage condition of −20°C is most suitable for conservation of the bacterial seed microbiome, as this storage temperature slows down the loss of seed bacterial diversity over longer time periods, particularly low-abundance taxa.

## Introduction

Seed vaults play a significant role in facilitating the *ex situ* conservation of germplasm of a range of crop species, their closely associated crop wild relatives (CWRs), and other wild species (Hay and Probert, 2013). Globally, seed vaults preserve plant genetic diversity for research and plant-breeding activities for improving food and nutritional security (Asdal and Guarino, 2018). International standards are adapted by seed vaults for long-term seed storage. According to the standard method, seeds are first kept under drying conditions at 10–15% relative humidity and 10–15°C to achieve a seed moisture content of 3–7% followed by their storage at or below −18°C. This method has been identified to work well for seeds of many plant species known as orthodox seeds (Cochrane et al., 2007).

Crop seeds are known to transmit a plant-specific core microbiota (Berg and Raaijmakers, 2018). The seed-associated microbes are reported to have plant genotype specificity and can vertically transmit from one generation to the next plant generation. Horizontal transfer of the microbes can occur *via* their uptake from the surrounding environment (Johnston-Monje et al., 2016; Shade et al., 2017; Adam et al., 2018; Nelson, 2018). The seed microbiome is known to harbor a wide range of microbial species (Johnston-Monje et al., 2016; Shade et al., 2017; Adam et al., 2018). The seed-borne microbes can colonize the emerging seedlings before the intake of microbes from the surrounding environment and can promote germination and early plant vigor and survival (Truyens et al., 2015). However, how seed-associated microbes influence the different growth stages during seed germination and later plant growth and provide biotic–abiotic stress resistance still need to be investigated (Adam et al., 2018; Berg and Raaijmakers, 2018).

Different seed-associated microbes were identified to protect crops against various biotic–abiotic stresses and enhance plant growth (Links et al., 2014; Mousa et al., 2016; Gdanetz and Trail, 2017; Shahzad et al., 2018; Li et al., 2020, 2021; Hone et al., 2021). In last two decades, the use of growth-promoting bacteria in agriculture has increased significantly to reduce the use of chemical fertilizers and enhance plant nutrient uptake (Rascovan et al., 2016). It is suggested that embracing greater use of beneficial microorganisms can improve crop yield and encourage biology-based agriculture (Schmidt et al., 2015).

Soybean [*Glycine max* (L.) Merr.] is one of the most important crops and a major source of animal feed and vegetable oil worldwide (Sugiyama et al., 2015). Due to a high protein (40–42%) and oil content (18–22%), soybean is also used for aquaculture feed and production of biofuel and as a source of protein for the human diet (Pagano and Miransari, 2016). Soybeans can play an important role in matching the food demands of the growing population by 2050, although the estimated yield increase of only 1.3% per year is not satisfactory (Pagano and Miransari, 2016). Some bacterial genera including *Pseudomonas*, *Bacillus*, *Bradyrhizobium*, *Streptomyces*, *Rahnella*, and *Azospirillum* and fungi, e.g., *Piriformospora* and *Trichoderma*, have proven to be promising in plant growth promotion in soybean production (Tsavkelova et al., 2006; Ramírez and Kloepper, 2010; Schmidt et al., 2015; Bakhshandeh et al., 2020). Notably, many crop, plant, and vegetable seeds were also reported to be inhabited by some of these bacterial genera (Johnston-Monje et al., 2016; Liu et al., 2017; Adam et al., 2018; Berg and Raaijmakers, 2018; Khalaf and Raizada, 2018; López et al., 2018; Wassermann et al., 2019a; Abdullaeva et al., 2021; Hone et al., 2021). However, despite the enormous potential of seed microbiomes to promote plant growth and sustainable agricultural practices, the impact of current international seed storage strategies on the seed microbial diversity and composition has not yet been evaluated (Berg and Raaijmakers, 2018).

Therefore, in the present study, amplicon sequencing of the V4 region of 16S rRNA was used to examine the impact of standard storage methods on the diversity and composition of the *G. max* seed bacterial microbiome and bacteria isolated from these seeds. The aim was to determine the effectiveness of current storage methods in maintaining the original bacterial composition and viability of seed-associated bacteria over time, thus providing an experimental basis for our understanding of the implication of seed vault storage strategies in conservation of seed bacterial microbiome.

## Materials and Methods

### Soybean Seed Samples

Soybean seeds (*G. max*-Burrinjuck) used in this study were sourced from Australian Grains Genebank, Horsham, Australia. About 1kg of seed was placed in a cloth bag and stored in a drying chamber at 15°C and 15% relative humidity for about 1month. This is the standard drying protocol used prior to storage of seed in the seed vault (FAO, 2014). At the end of the drying phase, about 100g of seed were weighed and transferred into heat-sealed aluminum bags. One bag of undried seed was also prepared to use it as control for the drying process at 0 time point. The seed bags were then transferred after 2days to the laboratory in AgriBio, Bundoora, Victoria, Australia. Seed bags containing dried seed were then stored at −20°C (±2°C), at 4°C (±2°C), and at room temperature (RT), 22°C (±2°C), for 3, 6, and 14months. Seed bags for RT were kept in an airtight plastic container at RT for 3, 6, and 14months. For further study, germinated seedlings were selected for profiling the *G. max* microbiome to focus on the seed-borne microbes that can colonize seedlings during germination, with the hypothesis that these microbes have a function in this process, thus focusing only on the viable microbial communities that remain after storage.

### Seed Germination

For germination, *G. max* seeds for each time point and storage temperature were washed 10 times with an excess amount of sterile distilled water. The seeds were transferred into sterile petri dishes (12-cm diameter) by placing them between pre-water soaked Whatman™ filter paper (two sheets underneath and one on top) and then sealed with Parafilm™ and kept in darkness for 24–32h at RT. Then, the top layer of filter paper was removed, and the plates were resealed with Parafilm™ followed by a further 8–10days of incubation on a lab-benchtop under ambient light conditions. Non-germinated seeds were discarded immediately to avoid any antagonistic fungal outgrowth from these seeds. If needed, water was sprayed on seedlings during the incubation under sterile conditions. The average germination rate for the *G. max* seeds remained between 55 and 60% during the study. Seedlings were harvested for microbiome profiling and microbial isolation once the cotyledons reached an unfolded growth stage (Supplementary Figure S1).

### DNA Extraction, 16S Amplicon Library Construction, and Sequencing

For seed microbiome profiling, 15–20 seedlings were selected for DNA extraction. Whole seedlings (root, shoot, and cotyledon) were cut into pieces of approximately 0.5–1cm using a sterile scalpel blade, collected in 1.2-ml QIAGEN collection tubes, and snap-frozen in liquid nitrogen and stored at −80°C until processed for DNA extraction. DNA extraction was performed using the MagAttract® 96 DNA plant kit using a Biomek FXᴾ Lab Automation Workstation coupled to a Synergy 2 multi-mode reader controlled by Biomek software version 4.1 and Gen 5 (2.08) software (Biotek Instruments, United States) with slight changes in manufacturer’s guidelines.

Amplicon libraries for Illumina sequencing were prepared using barcoded primer 5151f-806r, specific to V3–V4 regions of the bacterial 16s rRNA gene. Amplification of the host chloroplast and mitochondrial 16s DNA was blocked by adding peptide nucleic acids, pPNA and mPNA, respectively, to the PCR mix (Lundberg et al., 2013). PCR for 16s rRNA gene amplification was performed in a total volume of 25μl [Kapa HiFi Hotstart 2× ReadyMix DNA polymerase (Kapa Biosystems Ltd., London, United Kingdom), 50μM of pPNA and mPNA mix, 5μM of each primer, PCR-grade water, and 5μl of template DNA] under the following cycling conditions: 94°C for 3min, 30cycles of 94°C for 15s, 75°C for 10s, 55°C for 10s, 72°C for 45s, and a final elongation at 72°C for 10min using a thermal cycler (Agilent SureCycler 8,800, Agilent Technologies, United States). Libraries were further purified using AMPure XP beads (LABPLAN, Naas, Ireland). Dual indices and Illumina sequencing adapters from the Illumina Nextera XT index kits v2 B and C (Illumina, San Diego, United States) were added to the target amplicons in a second PCR step using Kapa HotStart HiFi 2× ReadyMix DNA polymerase (Kapa Biosystems Ltd., London, United Kingdom). Cycle conditions were 95°C (3min), then 10cycles of 95°C for 30s, 55°C for 30s, 72°C for 30s, then a final extension of 72°C for 5min followed by library cleanup using AMPure XP beads.

The barcoded libraries were quantified on a Nanodrop™ 1000 spectrophotometer and pooled together in an equimolar concentration. Library pools were further quantified for concentration and size using QuantiFluor® dsDNA assay (Promega Corporation, United States) and Tape station 2,200 High Sensitivity D1000 kit (Agilent Technologies, United States), respectively. Paired-end sequencing was performed on Miseq v3 (2×300bp v3 chemistry cartridge). All Illumina sequences have been submitted to the NCBI Sequence Read Archive (SRA accession PRJNA766782).

### Data Analysis

The raw Illumina® paired-end reads were quality filtered and merged into a single read using PEAR with default parameters (Zhang et al., 2014). Afterward, sequencing data analysis was performed using QIIME 22020.11.1 (Bolyen et al., 2019). The primers from single-end reads were then removed using cutadapt plugin with the following parameters; error rate-0.2, flags; adapter-wildcards, read-wildcards, and discard-untrimmed (Martin, 2011). The single-end reads were then trimmed to a read length of 253bp and then dereplicated and filtered to remove chimeras. A feature table was then constructed containing the amplicon sequencing variants (ASVs) and representative sequences using the default algorithm in DADA2 (Callahan et al., 2016). The ASVs were then aligned with mafft (Katoh et al., 2002; *via* q2-alignment) and used to construct a phylogeny tree with fasttree2 (Price et al., 2010; *via* q2-phylogeny). The taxonomic classification of ASVs was performed using a naive Bayes taxonomy classifier (Bokulich et al., 2018) trained on the silva-138 release (V4 region-16s rRNA gene; Quast et al., 2013). Host-associated mitochondria and chloroplast reads and low-abundance features (<10 counts and present in at least two samples) were discarded from the data using the filter-features plugin. Alpha diversity (Observed OTUs) and beta diversity (Jaccard distance) were explored by running the core-metrics script in QIIME2 by rarefying feature tables to a read count of 6,000 sequences. The taxa classified up to the genus level were then exported and used to perform the presence/Absence test in Genedata Expressionist® Analyst™ v.10.0 using the default parameters to identify the shared features between undried seed and dried seed stored for 0, 3, 6, and 14months at RT, 4, and −20°C (Genedata, Basel, Switzerland). Statistical analyses of the 16s rRNA gene data were performed using scripts in QIIME2 2020.11.1. Alpha diversity was tested for significant differences using the Kruskal–Wallis pairwise test and Beta diversity using Analysis of Similarity (ANOSIM) test. To determine the changes in bacterial abundance after seed drying, the QIIME2 feature table was exported in biom format and one-way ANOVA test was performed on individual ASVs (>0.1% *in planta*) using OriginPro 2019 (v9.6.0.172).

### Microbial Isolation, DNA Extraction, 16S Amplicon Library Construction, and Sequencing

For all storage temperatures, seedlings in triplicates were harvested by collecting the shoot and root tissues and discarding the seed coat. The plant tissues were cut into small pieces (0.5–1cm) and homogenized using a sterile pestle or two cycles of a Qiagen TissueLyser II for 1min at 30 Hertz in 400–500μl of 1× PBS buffer followed by centrifugation at 4,000rpm for 1min. Serial dilutions were prepared (10^−1^–10^−4^), and 20-μl aliquots, including undiluted macerate, were plated onto Reasoner’s 2A agar (R2A; Oxoid, United Kingdom), and the plates were incubated at RT for up to 10days. The bacterial growth was observed both in undiluted and diluted plates, though only undiluted plates were selected for further study to capture a snapshot of the original viable bacterial community composition that was culturable, as it is likely to contain the most diversity. The DNA was extracted by scraping all microbial colonies off the plate using a sterile plastic loop into an Eppendorf tube®. The colonies were then resuspended in 1× PBS buffer and spun at 10,000rpm for 5min. According to the manufacturer’s guidelines, the supernatant was discarded, followed by DNA extraction using Promega™ Wizard™ Genomic DNA Purification Kit (United States). The gDNA’s optical density measurements were performed in a Quantus™ Fluorometer (Promega Corporation, Madison, WI, United States), 16S rRNA bacterial genes were amplified, and libraries were prepared and sequenced using steps mentioned in Section DNA Extraction, 16S Amplicon Library Construction, and Sequencing.

### Data Analysis of Cultured Microbial Libraries

The raw paired-end reads were processed and analyzed as mentioned in Section Data Analysis, except the features were only filtered based on frequency (<10 counts), and the sequences were rarefied to the read counts of 16,529 sequences.

## Results

### 16S Amplicon Sequencing

After aligning paired-end reads, removal of low-frequency features, singletons, and chimeric and plant sequences, a total of 17,038,933 sequences were assigned to 361 Amplicon Sequence Variants (ASVs) for microbiome profiling (*in planta*) and 4,020,424 sequences were assigned to 155 ASVs for microbial isolation (on plates). After rarefaction and collapsing biological replicates, the ASV table was assigned to 89 genera for microbiome profiling (Supplementary Table S1) and 26 genera for microbial isolation (Supplementary Table S2).

### *G. max* Seed Microbiome Profiling (*In planta*)

**Figure 1:**
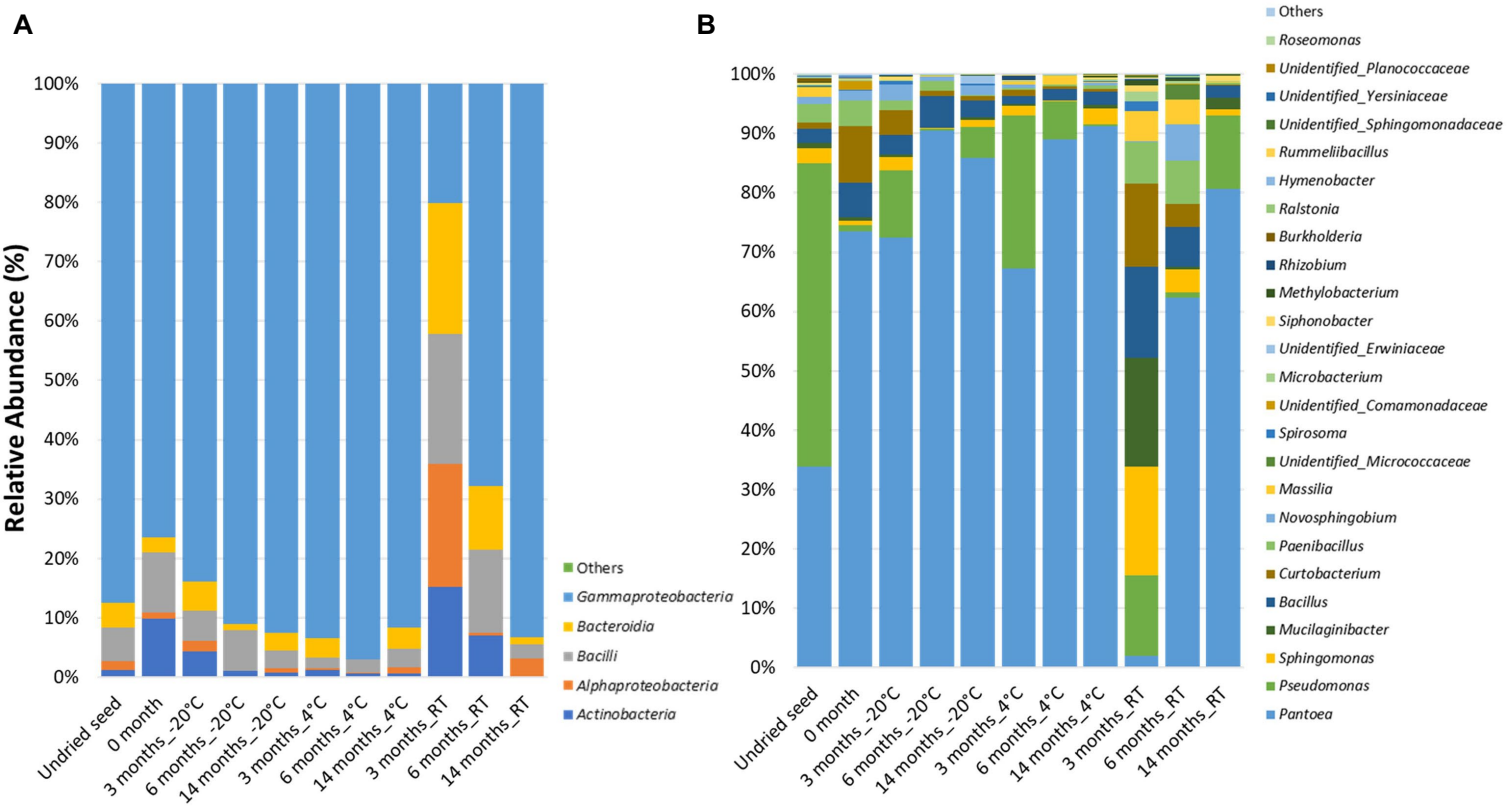
Relative abundance of the bacterial taxa *in planta* **(A)** at class level and **(B)** at genus level in undried seed and dried seed stored at different time points (0, 3, 6, and 14months) at −20°C, 4°C, and room temperature (RT). The bacterial taxa occurring with <0.1% are shown as “Others.”

At the class level, the bacterial communities were mainly dominated by the presence of *Gammaproteobacteria* (20.1–97.0%), *Bacilli* (1.8–21.9%), *Alphaproteobacteria* (0.1–22.1%), *Actinobacteria* (0.2–15.2%), and *Bacteroidia* (0.01–20.6%). Notably, after 3months at RT, the relative abundance was almost equally dominated by *Alphaproteobacteria* (22.1%), *Gammaproteobacteria* (20.1%), *Bacilli* (21.9%), *Bacteroidia* (20.6%), and *Actinobacteria* (15.2%). Additionally, low-abundance bacterial classes (<0.1%) such as *Polyangia* (0.0038%) and *Acidobacteriae* (0.00083%) designated as “Others” were observed in undried seed and seed stored for 3months at −20°C, respectively (Figure 1A, Supplementary Table S1).

At the genus level, 25 bacterial genera were identified with >0.1% relative abundance. The bacterial communities of the stored *G. max* seed mainly consisted of genera including *Pantoea* (1.8–91.2%), *Pseudomonas* (0.31–51.1%), *Bacillus* (1.4–14.9%), *Sphingomonas* (0.04–17.8%), *Curtobacterium* (0.17–13.7%), *Paenibacillus* (0.15–7.2%), *Mucilaginibacter* (0.0–17.8%), *Novosphingobium* (0.001–6.2%), and *Massilia* (0.01–4.9%) (Figure 1B). In addition to the community structure changes, the relative abundance of bacterial genera varied across all time points and storage temperatures. Interestingly, high variations in bacterial abundance were observed after 3months in seed stored at RT. In particular, majority of the ASVs mainly belonged to the genera *Sphingomonas* (20.7%), *Mucilaginibacter* (17.8%), *Bacillus* (14.9%), and *Curtobacterium* (13.7%), which were represented at much higher levels than at other time points (Figure 1B). Notably, the relative abundance of *Pantoea* (1.9%) was the lowest after 3months at RT compared to all other time points. The bacterial genera with >10% relative abundance *in planta* such as *Pantoea*, *Pseudomonas*, *Mucilaginibacter*, *Bacillus*, and *Curtobacterium* responded differently to drying treatment and storage temperature. It was observed that the relative abundance of *Pantoea* increased under cold storage, while *Pseudomonas* oscillates in abundance across all temperatures (Figure 1B, Supplementary Figure S2A). The bacterial genera with <10 and>1% relative abundance *in planta* such as *Paenibacillus*, *Novosphingobium*, *Massilia*, *Microbacteriaceae*, *Spirosoma*, *Microbacterium*, *Siphonobacter*, Unidentified group of *Comamonadaceae*, and *Erwiniaceae* were increased in abundance at RT especially after 3 and 6months of storage (Figure 1B, Supplementary Figure S2B). On the other hand, the bacterial genera with <1% such as *Methylobacterium*, *Rhizobium*, *Burkholderia*, *Ralstonia*, *Hymenobacter*, *Rummeliibacillus*, *Roseomonas*, Unidentified group of *Sphingomonadaceae*, *Yersiniaceae*, and *Planococcaceae* showed more variations in their abundance at 4°C and RT (Figure 1B, Supplementary Figure S2C).

### Composition Differences in the Seed Microbial Communities After Different Lengths of Storage

**Figure 2:**
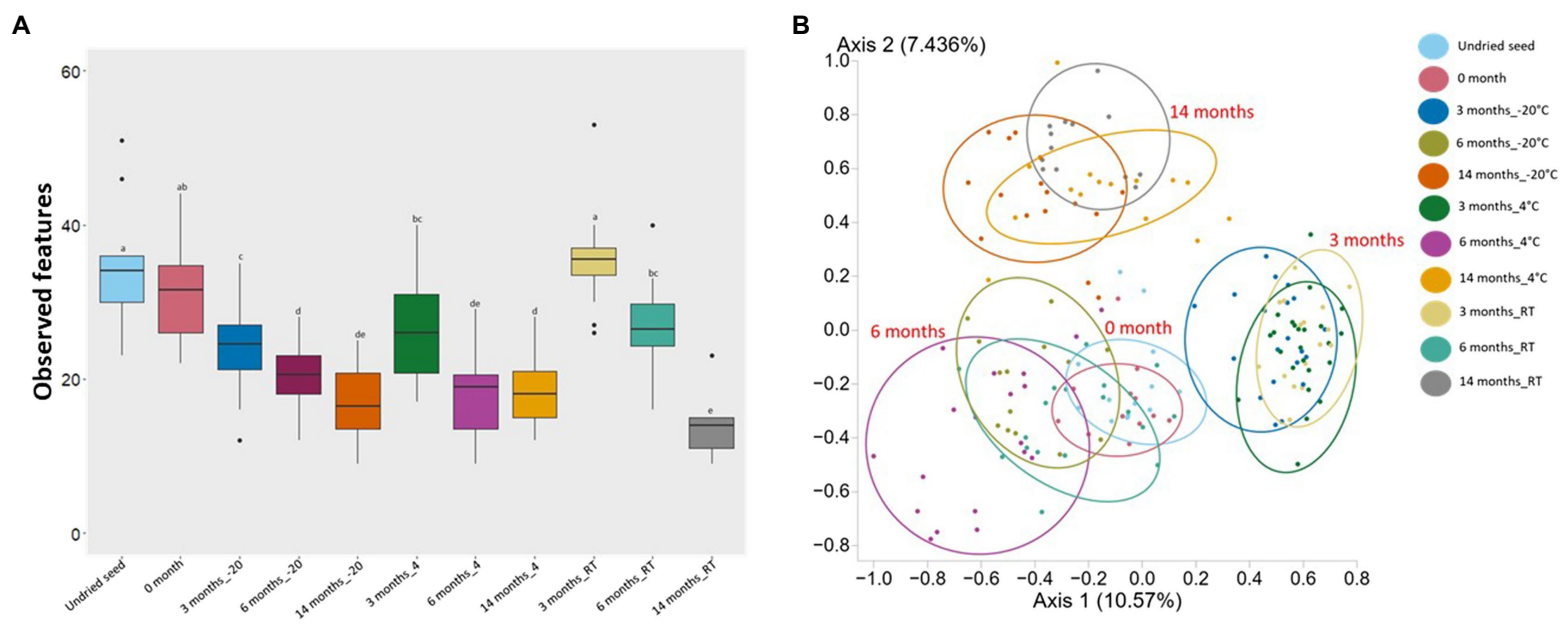
Alpha diversity (Observed features) and Beta diversity analysis (Jaccard dissimilarity) of the *G. max* seed (*in planta*). **(A)** Box-and-whisker plots showing the number of features observed under all conditions. Significant differences (*p*≤0.05) were assessed by ANOSIM pairwise test and are indicated by different lowercase letters (Supplementary Table S3). **(B)** Principal Coordinates Analysis (PCoA) plot showing the distances in the bacterial communities between undried seed and dried seed (0, 3, 6, and 14months) stored at −20°C (±2°C), 4°C (±2°C), and room temperature (RT; 22±2°C). Significant differences in bacterial composition were tested using the ANOSIM pairwise test (Supplementary Table S3).

Alpha diversity and beta diversity were used to assess the changes in bacterial diversity and composition in undried seed, dried seed, and seed stored for 0, 3, 6, and 14months at −20°C, 4°C, and RT. Based on alpha diversity, when compared to the undried seed, the number of observed features significantly (*p*<0.05) declined in dried seed stored at −20°C and 4°C in all time points (3, 6, and 14months) and in dried seed stored at RT for 6 and 14months. In contrast, no significant differences were observed between undried seed and dried seed (0month) and between undried seed and dried seed stored at RT for 3months. Additionally, when differences were compared for each storage temperature, the number of observed features significantly (p<0.05) declined after 3months and 6, 3, and 14months, in all time points at −20°C, 4°C, and RT, respectively (Figure 2A, Supplementary Table S3). No significant differences were observed for seed stored for 3 and 14months at −20 and 4°C, respectively.

Based on the Jaccard dissimilarity metrics, the bacterial composition significantly (p<0.05) varied under all conditions (Supplementary Table S3). These differences were also evident in the Principal Coordinates Analysis (PCoA) of the bacterial communities, where undried seed, dried seed (0month), and stored seed (3, 6, and 14months) formed a separate cluster (Figure 2B). Interestingly, the undried seed, dried seed (0month), and seed stored for 6months formed a close cluster compared to seed stored for 3 and 14months. The statistical analysis based on the presence/absence test was performed in Genedata Expressionist® Analyst™, and the number of shared genera was calculated between undried seed and dried seed (0month) and between undried seed and stored seed (3, 6, and 14months). There were 68.8% genera (33) shared between undried seed and dried seed (0month). While after 3months of storage, there were 56.3% genera (27) shared between undried seed and seed stored at RT, followed by 54.2% genera (26) with seed stored at 4°C and 47.9% genera (23) with seed stored at −20°C. Remarkably, after 6months of storage, there were 72.9% genera (35 genera) shared between undried seed and seed stored at RT followed by 64.6% (31) with seed stored at 4°C and 54.2% genera (26) with seed stored at −20°C. Next, after 14months of storage, there were 45.8% genera (22) shared between undried seed and seed stored at 4°C followed by 43.8% genera (21) seed stored at −20°C and 35.4% genera (17) seed stored at RT (Supplementary Figure S3).

### Culturability of the *G. max* Seed Bacterial Microbiome (On Plates)

**Figure 3:**
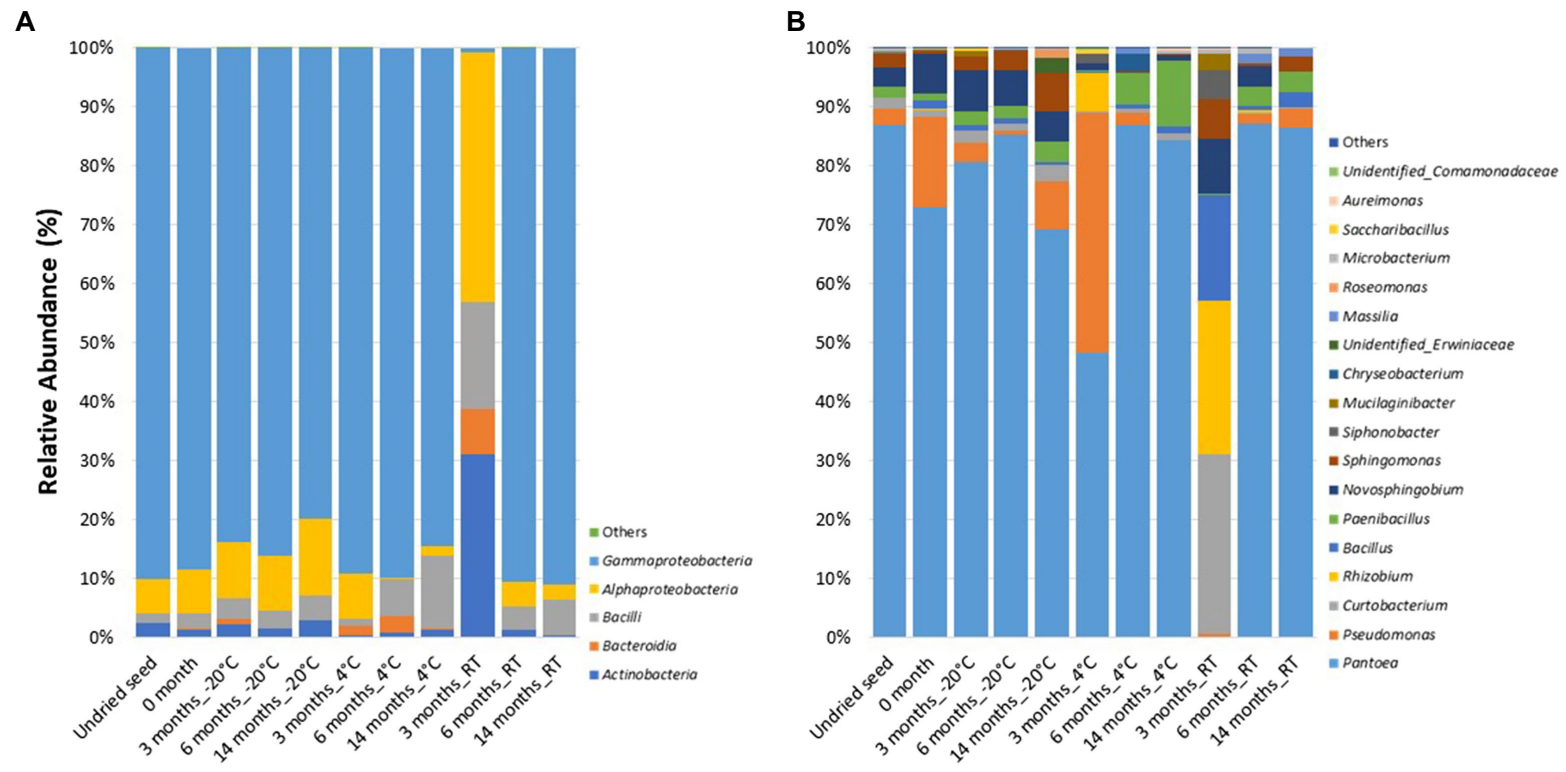
Relative abundance of bacterial taxa on plates **(A)** at class level and **(B)** at genus level in undried seed and dried seed stored at different time points (0, 3, 6, and 14months) at −20°C, 4°C, and room temperature (RT). The bacterial taxa occurring with less than 0.1% are shown as “Others.”

At the class level, the bacterial communities were mainly dominated by the presence of *Gammaproteobacteria* (0.7–91.1%), *Alphaproteobacteria* (0.3–42.4%), *Bacilli* (1.2–18.1%), *Actinobacteria* (0.3–31%), and *Bacteroidia* (0.0–7.8%). In contrast to the microbiome profiling (*in planta*), after 3months at RT (on plates), the relative abundance of bacterial classes was dominated by *Alphaproteobacteria* (42.4%), followed by *Actinobacteria* (31%), *Bacilli* (18.1%), *Bacteroidia* (7.8%), and *Gammaproteobacteria* (0.7%) (Figure 3A).

At the genus level, 18 bacterial genera were identified with >0.1% relative abundance. The bacterial communities on plates mainly consisted of genera *Pantoea* (0.2–87.3%), followed by *Pseudomonas* (0.0–40.7%), *Curtobacterium* (0.3–30.4%), *Rhizobium* (0.0–26.1%), *Bacillus* (0.01–17.9%), *Paenibacillus* (0.2–11.2%), *Novosphingobium* (0.0–9.3%), *Sphingomonas* (0.004–6.7%), *Siphonobacter* (0.0–4.8%), and *Mucilaginibacter* (0.0–3.0%). The relative abundance of these bacterial genera varied across all the time points and storage temperatures (Figure 3B). Similar to the microbiome profiling (*in planta*), a dramatic change in the relative abundance of bacterial genera was observed after 3months at RT (on plates). The distribution of abundance of these was different from that of the “*in planta*” data, with the most abundant bacteria belonging to *Curtobacterium* (30.4%), *Rhizobium* (26.1%), *Bacillus* (17.9%), *Novosphingobium* (9.3%), *Sphingomonas* (6.7%), *Siphonobacter* (4.8%), and *Mucilaginibacter* (3.0%). However, the relative abundance of *Pantoea* (0.2%) was lesser than the other time points, similar to *in planta* data (Figure 3B).

**Figure 4:**
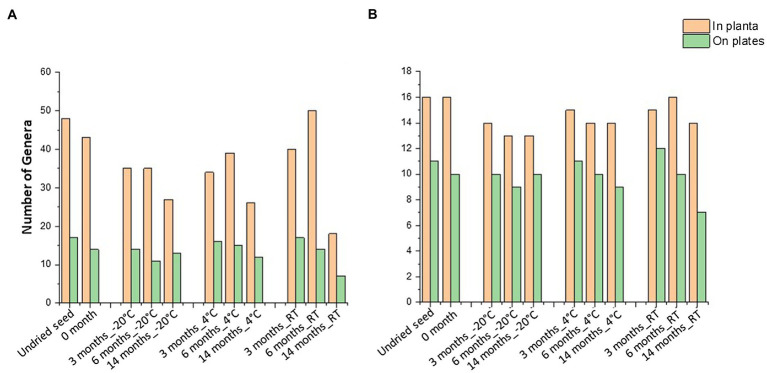
Culturable seed bacterial microbiome. **(A)** Number of genera observed *in planta* and on plates. **(B)** Number of genera with >0.1% abundance in undried seed and their culturability over time at different storage temperatures. All the seed samples belong to the undried seed, dried seed (0month), and seed stored for 3, 6, and 14months at −20°C, 4°C, and room temperature (RT).

When compared to the 16S rRNA gene sequencing data (*in planta*), not all the bacterial taxa (at genus level) were culturable. The overall culturability of the bacterial genera was more consistent at −20°C storage compared to the seed stored at 4°C and RT (Figure 4A). Many of the bacterial genera present with >0.1% relative abundance in undried seed (*in planta*) were culturable, with −20°C providing a more stable recovery compared to 4°C and RT after 14months of storage (Figure 4B).

**Figure 5:**
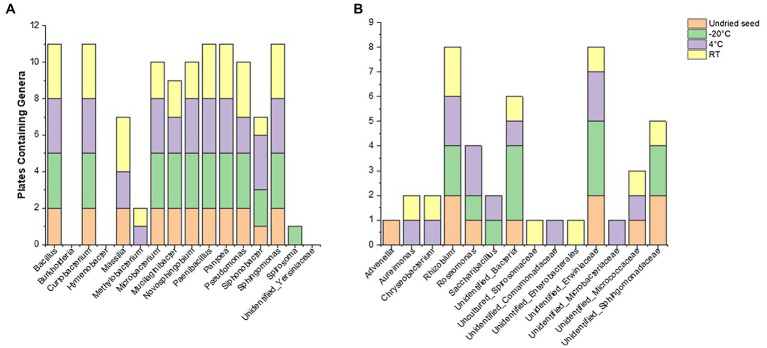
Culturability of bacterial genera that were associated with undried seed (*in planta*) across different time points (0, 3, 6, and 14months) when stored at −20°C, 4°C, and room temperature (RT). **(A)** Genera with >0.1% relative abundance and **(B)** <0.1% relative abundance in undried seed (*in planta*).

In total, 48 genera were associated with undried seed (*in planta*), of which 27 genera were culturable under the conditions of this experiment. There were 16 genera present with >0.1% relative abundance in undried seed (Supplementary Table S1), of which 13 genera were culturable with the exceptions being *Burkholderia* (0.6%), *Hymenobacter* (0.2%), and *Unidentified*_*Yersiniaceae* (0.1%) (Figure 5A). There were only five bacterial genera including *Pantoea*, *Sphingomonas*, *Bacillus*, *Curtobacterium*, and *Paenibacillus* detected on plates under all conditions. Notably, these were also some of the abundant genera *in planta*. While the presence of other bacterial genera on plates varied across all time points and storage temperatures (Figure 5A).

Of note, the abundance of *Massilia* declined when stored at −20°C *in planta* and was also not detected on plates from these seeds, showing its sensitivity to cold storage conditions (Figure 5A, Supplementary Figure S4). It was observed that some of the low-abundance bacterial genera (<0.1%) were also culturable including some unidentified bacteria (Figure 5B, Supplementary Table S2). On the other hand, some genera that were below the level of detection in undried seed and dried seed (0month) *in planta* including *Advenella*, *Aureimonas*, *Chryseobacterium*, *Uncultured_Spirosomaceae* and *Saccharibacillus* were detected on plates from varying storage temperatures (Figure 5B).

### Effect of Drying on the *G. max* Seed Microbiota

**Figure 6:**
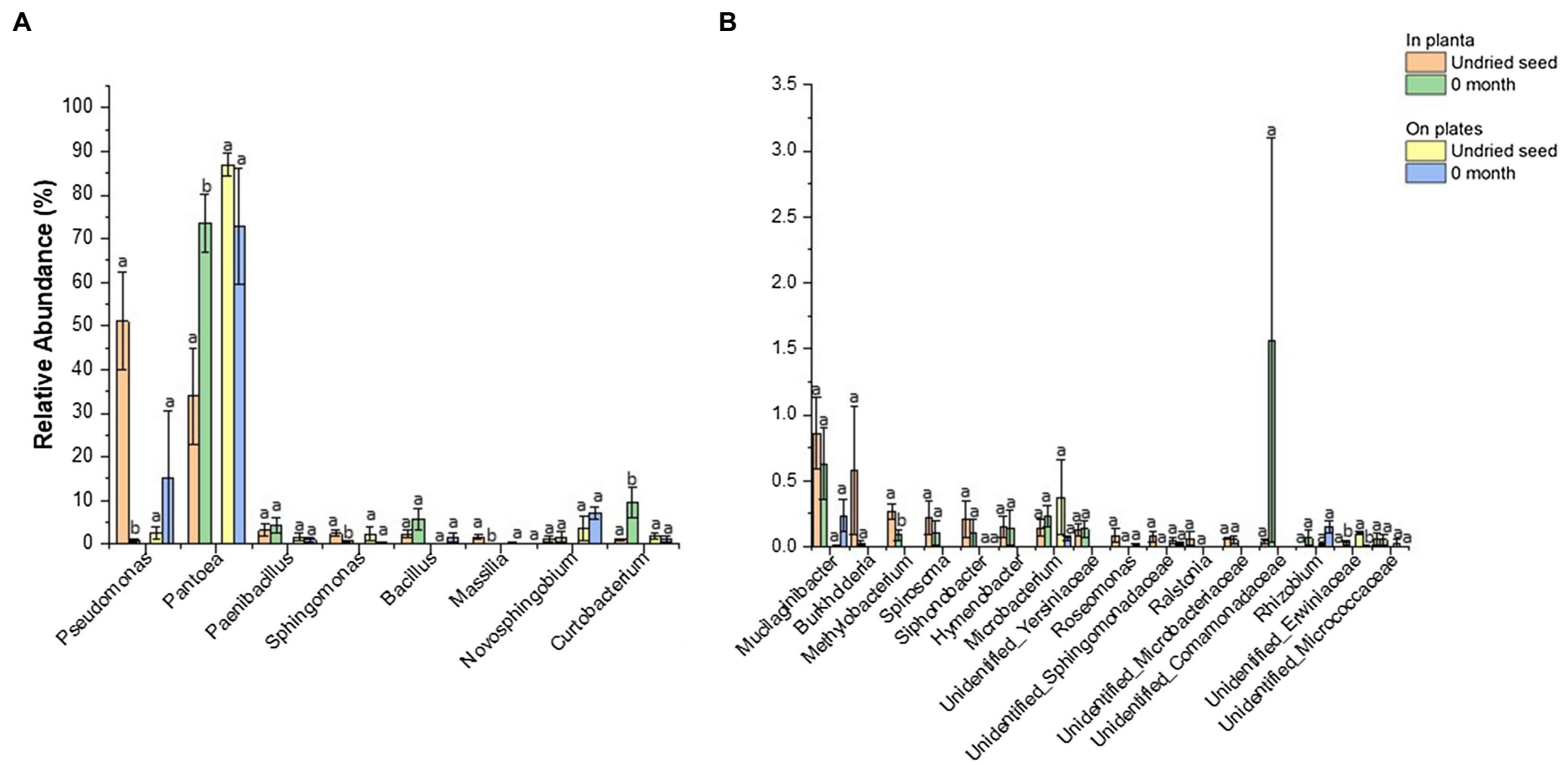
Comparison of relative abundance of bacterial genera detected in undried seed and dried seed (0month) *in planta* and on plates dataset. The abundance threshold was set based on *in planta* dataset (**A**) >1% and (**B**) <1% and >0.1% for statistical analysis. Significant differences (p≤0.05) were determined by one-way ANOVA test and are indicated by different lowercase letters. Error bars show SE of mean (SEM).

The drying treatment of *G. max* seed at 15°C and 15% relative humidity for 1month was found to alter the relative abundance of bacterial genera. The seed microbiome profiling (*in planta*) showed a significant change in the abundance of bacterial genera after drying including *Pseudomonas* (51.1 to 0.9%), *Pantoea* (33.9 to 73.6%), *Curtobacterium* (1.0 to 9.5%), *Sphingomonas* (2.5 to 0.7%), *Massilia* (1.6 to 0.005%), *Methylobacterium* (0.3 to 0.09%), and *Unidentified_ Erwiniaceae* (0 to 0.03%) (Figure 6A, Supplementary Table S5). There were four low-abundance genera including *Pajaroellobacter*, *Nesterenkonia*, env.OPS_17, and *Acidibacter* that completely disappeared after seed drying (Supplementary Table S1).

In contrast, no significant differences were observed for bacterial abundance on plates except for *Unidentified_ Erwiniaceae* (0.1 to 0.004%) (Figure 6B, Supplementary Table S5). The culturing assays, however, showed a different pattern for *Pseudomonas* (2 to 15%) and *Pantoea* (86.9 to 73.0%). It was observed that one replicate plate for dried seed (0month) was equally dominated by *Pantoea* (47.0%) and *Pseudomonas* (45.7%), contributing to an increased abundance of *Pseudomonas* (15.3%) and decreased abundance of *Pantoea* (73.0%) on plates post drying, indicating that the microbial diversity could vary from seed to seed to some extent (Figure 6B, Supplementary Table S4).

## Discussion

Seed banks have been created to preserve plant and crop genetic diversity for future use. However, the value of current seed storage techniques in conserving seed-borne microbial diversity has not been investigated (Berg and Raaijmakers, 2018). In this study, we demonstrated that drying *G. max* seed before storage changes the abundance and composition of the seed microbiota. Moreover, we found that the different bacterial communities respond differently to the seed drying and storage temperature. Additionally, the seed bacterial composition changed more dramatically under RT storage than the cold storage (−20 and 4°C). The culturability of seed-associated microbes was found to be largely driven by abundance. Seed storage at −20°C provided a long-term stable recovery of the culturable microbes under the conditions of this experiment.

### *G. max* Seed Microbiome Composition

Soybean seeds are known to lose viability and vigor under high temperature and relative humidity conditions (Shelar et al., 2008). Nevertheless, in this study, the impact of such environmental factors was reduced due to the optimal and stable conditions during a 14-month storage period. In general, the *G. max* seed microbiome consisted of bacterial classes such as *Gammaproteobacteria*, *Alphaproteobacteria*, *Bacilli*, *Bacteroidia*, and Actinobacteria. Previous studies also observed these bacterial classes for the seed of red sage (Chen et al., 2018), bean (Klaedtke et al., 2016), ryegrass (Tannenbaum et al., 2020), rice (Nakaew and Sungthong, 2018), native alpine plants (Wassermann et al., 2019a), and *Brassicaceae* family plants (Barret et al., 2015). It has been demonstrated that the microbes colonizing seedling during germination can confer important functional traits to the plant such as nutrient availability (Torres-Cortés et al., 2018). Representatives of the majority of the detected bacterial genera including *Pantoea*, *Pseudomonas*, *Bacillus*, *Sphingomonas*, *Curtobacterium*, *Paenibacillus*, *Mucilaginibacter*, *Novosphingobium*, and *Massilia* are known to have a beneficial impact on plants. For instance, *Bacillus* strains isolated from soybean root nodules have been observed to promote soybean plant growth when co-inoculated with *Bradyrhizobium japonicum* under nitrogen-free conditions (Bai et al., 2003). Also, some *Pseudomonas* species isolated from the soybean rhizosphere inhibited the growth of soilborne pathogenic fungi (Susilowati et al., 2011). However, other species of *Pseudomonas* are well known for their pathogenicity (Xin et al., 2018; Solanki et al., 2019). The endophytic bacteria from the soybean root nodules were also identified to contain plant growth-promoting traits and antagonistic properties against pathogenic fungi (*Phytophthora sojae*; Zhao et al., 2018).

### Effect of Seed Drying on *G. max* Seed Microbiome Composition

Seed banks globally follow seed drying treatment prior to low-temperature storage to increase seed longevity (Hay and Probert, 2013). While this treatment is valuable for increasing seed longevity in storage, there is little evidence about the effect of seed drying on seed microbiome conservation. In this study, both the seed drying and storage temperature were identified to affect the seed bacterial composition. Some of the abundant bacteria, including *Pseudomonas*, *Sphingomonas*, *Massilia*, *Curtobacterium*, and *Methylobacterium* declined significantly after seed drying treatment. This change in composition corresponded to a significant increase in abundance of *Pantoea* from an average of 33.9% to more than 73%, with four bacterial genera remaining undetected. It was recognized that temperature, humidity, water activity, and grain moisture could affect the seed microbial community (Schmidt et al., 2018). It must be stated that the bacterial communities can respond differently to the water stress caused during the seed drying process (Esbelin et al., 2018). The exclusive increase in the abundance of *Pantoea* after seed drying may indicate their ability to tolerate the stress caused due to water loss. *Pantoea* spp. along with *E. sakazakii*, *E. vulneris*, and *K. oxytoca* were reported to persist over 2years when individual bacterial strains were subjected to desiccated storage. This ability was attributed to the formation of an extracellular polysaccharide that can facilitate the survival of bacterial strains during an extended desiccation period (Lehner et al., 2005; Barron and Forsythe, 2007). Notably, the results obtained with culturing assays of microbes isolated from undried seed and dried seed (0month) were in general agreement with results obtained by culture-independent 16S rRNA gene sequencing data obtained *in planta*. However, an opposite pattern was observed for the relative abundance pattern of *Pantoea* and *Pseudomonas* between the culturing assay and the *in planta* assay. Interestingly, it was identified that the differences in bacterial abundance among seed samples were responsible for this variation. This was also in line with previous studies that have identified that relative abundance of bacterial inhabitants of seed can vary significantly between seed samples of the same plant species (Barret et al., 2015; Klaedtke et al., 2016; Rybakova et al., 2017; Rezki et al., 2018; Torres-Cortés et al., 2018). It has been reported that other than plant genotype, abiotic factors such as storage, harvesting methods, and field management practices were identified as possible drivers of such variations in bacterial abundance among seed samples (Barret et al., 2015).

### Effect of Storage Temperature on *G. max* Seed Microbiome Composition

In the present study, *Gammaproteobacteria* dominated the *G. max* seed bacterial microbiome under all conditions. Notably, this was due to an increased abundance of *Pantoea* after seed drying compared to other abundant genera including *Pseudomonas*, *Sphingomonas*, *Mucilaginibacter*, *Bacillus*, and *Curtobacterium*. Endophytic strains of *Pantoea* isolated from the surface-sterilized leaves of *Alhagi sparsifolia Shap*. and wheat roots have been shown to improve plant growth under drying conditions (Chen et al., 2017; Cherif-Silini et al., 2019). A strain of *Pantoea dispersa* (Selvakumar et al., 2008) isolated from a sub-alpine soil was able to grow under different temperature conditions ranging from 4 to 42°C. Notably, more variations in bacterial abundance were observed in seed stored at RT. For instance, after 3months of storage at RT, the abundance of genera including *Sphingomonas*, *Mucilaginibacter*, *Bacillus*, and *Curtobacterium* collectively accounted for about 67% of the total bacterial abundance, which was higher than that at other time points. Interestingly, this change corresponded with a significant decrease in *Pantoea* from an average of 62.6% in undried seed and dried seed (0month) and seed stored for 6 and 14months to only 1.8% after 3months at RT. The bacterial strains belonging to the genera *Sphingomonas*, *Mucilaginibacter*, *Curtobacterium*, and *Bacillus* were identified to be more tolerant to drying conditions (Mannisto et al., 2010; Vardharajula et al., 2011; Chimwamurombe et al., 2016; Molina-Romero et al., 2017). Thus, it is highly plausible that these bacterial strains might be benefited by the sudden environmental changes created by seed drying and packaging. It must be stated that the majority of these genera are either aerobic or facultative anaerobes. It is highly possible that a reduction in available oxygen level due to an airtight heat-sealed packaging along with the drying conditions promoted favorable conditions for these genera. Moreover, this study showed that such changes in abundance were only for a limited period and the bacterial communities were more similar to the pre-drying conditions after 6months in all storage temperatures. Šťovíček et al. (2017) in their study showed that certain anaerobic taxa in soil dominated after a rainfall, but after 5–7days, the microbial composition returned to the pre-rainfall conditions. A similar trend was also reported by Supramaniam et al. (2016) who reported a shift in soil bacterial composition initially after a short variation in temperature and water content, though the bacterial composition was more similar to the control after 4weeks. A similar trend was also observed for the cultured bacterial taxa isolated from the stored *G. max* seed after 3months at RT, though the dominating bacterial genera varied compared to the *in planta* data. The differences in the bacterial abundance among individual seed samples were identified to contributing to these variations as observed for the seed drying in the above section (*Effect of Seed Drying on G. max Seed Microbiome Composition*). Notably, such variations in the abundance of bacterial genera at RT were not reflected in seed under cold storage.

We have shown that the seed bacterial diversity and composition co-vary with time and storage temperature. Results indicated that the seed can be stored at RT for 6months without losing much diversity and original bacterial composition. While the bacterial diversity and composition reduced rapidly after 6months. The disappearance of the lower-abundance bacterial genera was observed as the major reason for these diversity losses during storage. Seed storage at −20°C was identified as showing a gradual disappearance of the lower-abundance genera compared to more rapid losses at 4°C and RT. Soybean seed is known to go through various biochemical changes, such as decreases in fat, water-soluble nitrogen, sugars, nitrogen solubility index, trypsin inhibitor activity, available lysin, pigment, and lipoxygenase activity of seed and increases in seed browning, free fatty acid content, and peroxidase value when stored under ambient conditions (Narayan et al., 1988; Sharma et al., 2013). For instance, the increased level of free fatty acid in soybean seed invaded by *Aspergillus ruber* resulted in loss of seed viability (Dhingra et al., 2001). In our study, the significant decline in bacterial diversity and composition in *G. max* seed, specifically the loss of lower-abundance genera at RT and 4°C, might be linked to an increased level of free fatty acid. It has been identified that free fatty acid can kill bacteria by inhibiting enzyme activity, disrupting nutrient uptake, and lysing bacterial cells directly or indirectly (e.g., toxic peroxidation and autoxidation products; Desbois and Smith, 2010).

### Effect of Storage Temperature on the Culturability of *G. max* Seed Microbiome

Seed-associated microbes have the potential to promote plant growth and to provide sustainable ways to protect crops against various biotic and abiotic stresses in the form of seed treatments. Seed banks can play an important role in conservation of the beneficial seed-associated microbes. However, there is a need to design international conservation strategies for seed banks to protect seed microbes so that their untapped benefits for plant, human, and environment can be further explored in the future (Berg and Raaijmakers, 2018). Culturing of the seed microbes independently of stored seed has been suggested as a necessary step to ensure that the beneficial microbes remain available to use them for enhancing crop productivity (Rascovan et al., 2016; Sarhan et al., 2019). To understand the importance of independent microbe culturing, we decided to examine the impact of seed storage temperature on the culturability of microbes. Overall, the results obtained with culturing assay of the stored *G. max* seed agreed with results obtained by *in planta* 16S rRNA gene sequencing data. In the current study, the isolated bacteria mainly belonged to the *Gammaproteobacteria* and *Alphaproteobacteria*, with *Pantoea*, *Sphingomonas*, *Bacillus*, *Curtobacterium*, *Rhizobium*, and *Paenibacillus* being some of the dominant bacterial genera isolated from *G. max* seed throughout the study. Notably, most of these genera have been characterized with a range of beneficial features such as the ability to fix nitrogen, indole-3-acetic acid production, and antagonistic abilities against various bacteria and fungi (Silini-Cherif et al., 2012; Hansen et al., 2017; Goyal et al., 2019; Liu et al., 2019). Data indicated that the genera present with more than 0.1% relative abundance in undried seed remained culturable after 14months of storage under all conditions. Many of the bacterial genera identified by 16S rRNA gene sequencing data *in planta* were culturable; however, not all of them were identified in culturing assays. These results were consistent with previous findings, where majority of the microbes identified by sequencing were not detected using classical culturing approach (Sylla et al., 2013; Schmidt et al., 2014; Qaisrani et al., 2019; Solanki et al., 2019). Notably, the cold storage temperatures, especially −20°C storage provided a stable recovery for the bacterial genera present with greater than 0.1%, while the bacterial viability was adversely affected in seed stored at RT. Cabello-Olmo et al. (2020) investigated the effect of storage temperature and packaging on bacteria and yeast viability in a plant-based fermented food and indicated that the microbial content seemed to be better preserved at −20 and 4°C compared to storage at 37°C. We also observed that the genera *Massilia* was sensitive to cold storage. Especially at −20°C storage, their relative abundance gradually declined *in planta* and was poorly represented in the culturing assay. A significant reduction in the abundance of *Massilia* was also observed in apples after they were stored for 6months in a commercial cold storage. The cold sensitivity of *Massilia* was suspected as the main reason for the significant decline in abundance (Wassermann et al., 2019b).

In conclusion, our study demonstrated that standard storage methods can be used for conservation of seed-associated bacterial microbiome, especially for high-abundance genera. Given that seed drying significantly impacts the composition of *G. max* seed microbiome, we suggest that a fresh bacterial isolation can help to conserve the original bacterial composition. Moreover, −20°C storage has been identified as a better alternative to RT and 4°C, as the overall bacterial diversity losses including lower-abundance genera were reduced, and culturability rate was high in −20°C storage. A better understanding about the effect of the standard storage methods on the seed microbiome composition of different plant species including their wild relatives can assist in designing new international conservation strategies for seed microbiomes.

## Data Availability Statement

The raw sequence files supporting the findings of this article are available in the NCBI Sequence Read Archive (SRA) under the BioProject ID PRJNA766782.

## Author Contributions

TS conceptualized the study. AC prepared the article. AC, TS, JE, and RM designed the experiment. SN provided the soybean seed and helped with the seed drying. AC and JK contributed to the laboratory work. TS and RM reviewed and edited the article. TS, JE, and RM supervised the study. GS contributed to the funding acquisition. All authors have read and agreed to the submitted version of the article.

## Funding

This research was supported by the Agriculture Victoria Research.

## Conflict of Interest

The authors declare that the research was conducted in the absence of any commercial or financial relationships that could be construed as a potential conflict of interest.

## Publisher’s Note

All claims expressed in this article are solely those of the authors and do not necessarily represent those of their affiliated organizations, or those of the publisher, the editors and the reviewers. Any product that may be evaluated in this article, or claim that may be made by its manufacturer, is not guaranteed or endorsed by the publisher.
