# Corrigendum: Implications of Seed Vault Storage Strategies for Conservation of Seed Bacterial Microbiomes

**DOI:** 10.3389/fmicb.2022.913138

**Published:** 2022-04-25

**Authors:** Ankush Chandel, Ross Mann, Jatinder Kaur, Sally Norton, Jacqueline Edwards, German Spangenberg, Timothy Sawbridge

**Affiliations:** ^1^Agriculture Victoria Research, AgriBio, Centre for AgriBioscience, Bundoora, VIC, Australia; ^2^School of Applied Systems Biology, La Trobe University, Bundoora, VIC, Australia; ^3^Agriculture Victoria Research, Australian Grains Genebank, Horsham, VIC, Australia

**Keywords:** seed vault, storage strategies, seed bacterial microbiomes, conservation, culturability

In the original article, there was a mistake in Figure 1 as published. Incorrect color legend was assigned to the bar graph in Figure 1B. This has now been corrected. The corrected Figure 1 appears below.

**Figure 1:**
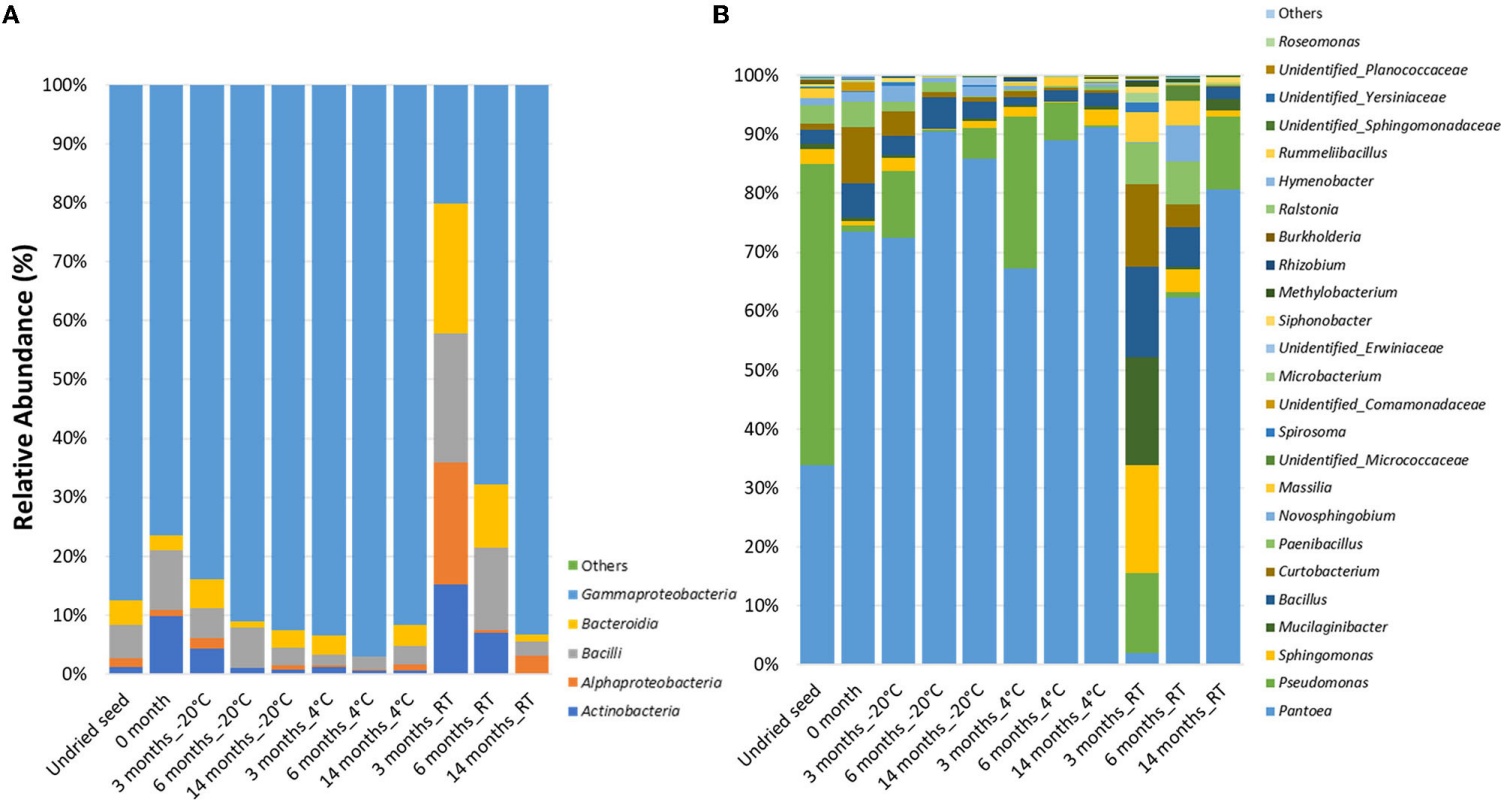
Relative abundance of the bacterial taxa *in planta* **(A)** at class level and **(B)** at genus level in undried seed and dried seed stored at different time points (0, 3, 6, and 14 months) at −20°C, 4°C, and room temperature (RT). The bacterial taxa occurring with <0.1% are shown as “Others”.

The authors apologize for this error and state that this does not change the scientific conclusions of the article in any way. The original article has been updated.

## Publisher's Note

All claims expressed in this article are solely those of the authors and do not necessarily represent those of their affiliated organizations, or those of the publisher, the editors and the reviewers. Any product that may be evaluated in this article, or claim that may be made by its manufacturer, is not guaranteed or endorsed by the publisher.

