# Supplementary material for: Implications of Seed Vault Storage Strategies for Conservation of Seed Bacterial Microbiomes
Source: Front Microbiol. 2021 Dec 3;12:784796. doi: 10.3389/fmicb.2021.784796 (PMC8678515; doi:10.3389/fmicb.2021.784796)
Supplement: Supplementary file 1 [file Data_Sheet_1.docx]

Supplementary Material

Figures and tables

# **Supplementary Tables**

**Table S1.** Relative abundance of the bacterial genera associated to G. max seed samples belonged to the undried seed, dried seed (0 month) and seed stored for 3-, 6- and 14 months at -20°C, 4°C and RT. Taxa occurring at >0.1% are highlighted in bold.

| **Taxonomic Level** | | | **Relative abundance (%)** | | | | | | | | | | | |  |
| --- | --- | --- | --- | --- | --- | --- | --- | --- | --- | --- | --- | --- | --- | --- | --- |
|  |  |  |  |  |  |  |  |  |  |  |  |  |  |  |  |
| **Phylum** | **Class** | **Genus** | **Undried seed** | **0 month** | **3 months_-20°C** | **6 months_-20°C** | **14 months_-20°C** | **3 months_4°C** | **6 months_4°C** | **14 months_4°C** | **3 months_RT** | **6 months_RT** | **14 months_RT** |  | |
|  |  |  |  |  |  |  |  |  |  |  |  |  |  |  | |
| *Unassigned_Bacteria* |  |  | 0.000 | 0.000 | 0.005 | 0.001 | 0.007 | 0.006 | 0.001 | 0.007 | 0.030 | 0.006 | 0.003 |  | |
| *Acidobacteriota* | *Acidobacteriae* | *Subgroup2* | 0.000 | 0.000 | 0.001 | 0.000 | 0.000 | 0.000 | 0.000 | 0.000 | 0.000 | 0.000 | 0.000 |  | |
| *Actinobacteriota* | *Actinobacteria* | *Corynebacterium* | 0.000 | 0.008 | 0.001 | 0.002 | 0.000 | 0.021 | 0.003 | 0.000 | 0.000 | 0.002 | 0.000 |  | |
| *Actinobacteriota* | *Actinobacteria* | *Lawsonella* | 0.000 | 0.000 | 0.000 | 0.000 | 0.000 | 0.000 | 0.000 | 0.000 | 0.011 | 0.002 | 0.000 |  | |
| *Actinobacteriota* | *Actinobacteria* | *Dietzia* | 0.000 | 0.000 | 0.000 | 0.000 | 0.000 | 0.000 | 0.000 | 0.000 | 0.000 | 0.003 | 0.000 |  | |
| *Actinobacteriota* | *Actinobacteria* | *Williamsia* | 0.000 | 0.000 | 0.000 | 0.000 | 0.000 | 0.000 | 0.000 | 0.000 | 0.000 | 0.002 | 0.000 |  | |
| *Actinobacteriota* | *Actinobacteria* | *Tsukamurella* | 0.000 | 0.000 | 0.000 | 0.000 | 0.000 | 0.000 | 0.001 | 0.000 | 0.000 | 0.000 | 0.000 |  | |
| *Actinobacteriota* | *Actinobacteria* | *Kineococcus* | 0.000 | 0.001 | 0.034 | 0.000 | 0.000 | 0.000 | 0.000 | 0.000 | 0.001 | 0.003 | 0.000 |  | |
| *Actinobacteriota* | *Actinobacteria* | *Quadrisphaera* | 0.005 | 0.000 | 0.013 | 0.001 | 0.000 | 0.000 | 0.000 | 0.001 | 0.000 | 0.006 | 0.000 |  | |
| *Actinobacteriota* | *Actinobacteria* | *Unidentified_Microbacteriaceae* | 0.004 | 0.026 | 0.000 | 0.019 | 0.000 | 0.000 | 0.006 | 0.000 | 0.000 | 0.002 | 0.003 |  | |
| *Actinobacteriota* | *Actinobacteria* | *Clavibacter* | 0.000 | 0.000 | 0.065 | 0.006 | 0.000 | 0.000 | 0.000 | 0.000 | 0.000 | 0.000 | 0.000 |  | |
| *Actinobacteriota* | *Actinobacteria* | *Curtobacterium* | **1.035** | **9.543** | **4.151** | **0.864** | **0.710** | **0.999** | **0.545** | **0.361** | **13.667** | **3.859** | **0.169** |  | |
| *Actinobacteriota* | *Actinobacteria* | *Frigoribacterium* | 0.000 | 0.022 | 0.000 | 0.000 | 0.000 | 0.000 | 0.000 | 0.000 | 0.001 | 0.000 | 0.000 |  | |
| *Actinobacteriota* | *Actinobacteria* | *Microbacterium* | **0.144** | **0.231** | **0.118** | 0.057 | 0.028 | **0.122** | 0.031 | **0.193** | **1.517** | **0.496** | 0.056 |  | |
| *Actinobacteriota* | *Actinobacteria* | *Rathayibacter* | 0.000 | 0.000 | 0.003 | 0.000 | 0.000 | 0.000 | 0.000 | 0.000 | 0.002 | 0.001 | 0.000 |  | |
| *Actinobacteriota* | *Actinobacteria* | *Unidentified_Micrococcaceae* | 0.060 | 0.053 | 0.000 | 0.000 | 0.000 | 0.001 | 0.004 | 0.002 | 0.008 | **2.530** | 0.000 |  | |
| *Actinobacteriota* | *Actinobacteria* | *Kocuria* | 0.000 | 0.000 | 0.000 | 0.004 | 0.000 | 0.000 | 0.000 | 0.000 | 0.000 | 0.000 | 0.000 |  | |
| *Actinobacteriota* | *Actinobacteria* | *Micrococcus* | 0.001 | 0.019 | 0.000 | 0.002 | 0.000 | 0.000 | 0.004 | 0.000 | 0.000 | 0.006 | 0.000 |  | |
| *Actinobacteriota* | *Actinobacteria* | *Nesterenkonia* | 0.003 | 0.000 | 0.000 | 0.000 | 0.000 | 0.000 | 0.000 | 0.000 | 0.000 | 0.000 | 0.000 |  | |
| *Actinobacteriota* | *Actinobacteria* | *Rothia* | 0.000 | 0.000 | 0.000 | 0.019 | 0.000 | 0.002 | 0.007 | 0.000 | 0.005 | 0.001 | 0.000 |  | |
| *Actinobacteriota* | *Actinobacteria* | *Cutibacterium* | 0.005 | 0.007 | 0.006 | 0.019 | 0.001 | 0.008 | 0.020 | 0.007 | 0.030 | 0.082 | 0.000 |  | |
| *Actinobacteriota* | *Actinobacteria* | *Propionibacterium* | 0.000 | 0.000 | 0.000 | 0.000 | 0.000 | 0.001 | 0.000 | 0.000 | 0.000 | 0.000 | 0.000 |  | |
| *Bacteroidota* | *Bacteroidia* | *Asinibacterium* | 0.000 | 0.000 | 0.000 | 0.000 | 0.002 | 0.000 | 0.000 | 0.000 | 0.000 | 0.000 | 0.000 |  | |
| *Bacteroidota* | *Bacteroidia* | *Sediminibacterium* | 0.000 | 0.001 | 0.000 | 0.000 | 0.000 | 0.000 | 0.000 | 0.000 | 0.000 | 0.000 | 0.000 |  | |
| *Bacteroidota* | *Bacteroidia* | *Siphonobacter* | **0.212** | **0.104** | **0.603** | 0.008 | 0.000 | 0.078 | 0.008 | **0.289** | **1.056** | 0.019 | **1.023** |  | |
| *Bacteroidota* | *Bacteroidia* | *Hymenobacter* | **0.150** | **0.144** | 0.000 | 0.000 | 0.021 | 0.020 | 0.000 | 0.011 | **0.193** | 0.009 | 0.003 |  | |
| *Bacteroidota* | *Bacteroidia* | *Dyadobacter* | 0.005 | 0.000 | 0.000 | 0.000 | 0.003 | 0.000 | 0.000 | 0.000 | 0.000 | 0.006 | 0.000 |  | |
| *Bacteroidota* | *Bacteroidia* | *Flectobacillus* | 0.000 | 0.000 | 0.000 | 0.000 | 0.008 | 0.000 | 0.000 | 0.000 | 0.000 | 0.000 | 0.001 |  | |
| *Bacteroidota* | *Bacteroidia* | *Spirosoma* | **0.218** | **0.104** | **0.598** | 0.000 | **0.272** | 0.026 | 0.000 | **0.206** | **1.606** | 0.054 | 0.004 |  | |
| *Bacteroidota* | *Bacteroidia* | *Flavobacterium* | 0.000 | 0.006 | 0.000 | 0.000 | 0.000 | 0.000 | 0.000 | 0.000 | 0.000 | 0.000 | 0.000 |  | |
| *Bacteroidota* | *Bacteroidia* | *Chryseobacterium* | 0.000 | 0.000 | 0.000 | 0.000 | 0.000 | 0.000 | 0.032 | 0.002 | 0.000 | 0.000 | 0.000 |  | |
| *Bacteroidota* | *Bacteroidia* | *Cloacibacterium* | 0.000 | 0.000 | 0.000 | 0.000 | 0.002 | 0.003 | 0.000 | 0.000 | 0.000 | 0.000 | 0.000 |  | |
| *Bacteroidota* | *Bacteroidia* | *Mucilaginibacter* | **0.858** | **0.628** | **0.491** | 0.000 | **0.481** | **0.217** | 0.055 | **0.531** | **17.783** | **0.382** | **1.949** |  | |
| *Bacteroidota* | *Bacteroidia* | *Sphingobacteriales_env.OPS17* | 0.006 | 0.000 | 0.000 | 0.000 | 0.000 | 0.000 | 0.000 | 0.000 | 0.000 | 0.000 | 0.000 |  | |
| *Firmicutes* | *Bacilli* | *Tumebacillus* | 0.000 | 0.000 | 0.043 | 0.000 | 0.000 | 0.000 | 0.000 | 0.000 | 0.000 | 0.000 | 0.000 |  | |
| *Firmicutes* | *Bacilli* | *Unidentified_Bacillales* | 0.017 | 0.000 | 0.000 | 0.000 | 0.005 | 0.000 | 0.000 | 0.000 | 0.013 | 0.013 | 0.000 |  | |
| *Firmicutes* | *Bacilli* | *Unidentified_Bacillaceae* | 0.001 | 0.003 | 0.000 | 0.000 | 0.000 | 0.000 | 0.000 | 0.000 | 0.000 | 0.000 | 0.000 |  | |
| *Firmicutes* | *Bacilli* | *Anaerobacillus* | 0.003 | 0.010 | 0.000 | 0.010 | 0.000 | 0.000 | 0.003 | 0.000 | 0.000 | 0.000 | 0.000 |  | |
| *Firmicutes* | *Bacilli* | *Bacillus* | **2.359** | **5.800** | **3.343** | **5.267** | **2.850** | **1.392** | **1.888** | **2.331** | **14.888** | **6.811** | **2.035** |  | |
| *Firmicutes* | *Bacilli* | *Unidentified_Planococcaceae* | 0.000 | 0.000 | 0.000 | 0.000 | 0.000 | 0.000 | 0.000 | 0.000 | **0.106** | 0.000 | 0.000 |  | |
| *Firmicutes* | *Bacilli* | *Lysinibacillus* | 0.000 | 0.000 | 0.004 | 0.000 | 0.000 | 0.000 | 0.000 | 0.000 | 0.000 | 0.000 | 0.000 |  | |
| *Firmicutes* | *Bacilli* | *Rummeliibacillus* | 0.000 | 0.000 | 0.007 | 0.000 | 0.000 | 0.013 | 0.000 | **0.171** | **0.189** | 0.000 | 0.000 |  | |
| *Firmicutes* | *Bacilli* | *Carnobacterium* | 0.014 | 0.014 | 0.000 | 0.002 | 0.000 | 0.000 | 0.021 | 0.000 | 0.000 | 0.014 | 0.000 |  | |
| *Firmicutes* | *Bacilli* | *Dolosigranulum* | 0.000 | 0.000 | 0.000 | 0.000 | 0.000 | 0.000 | 0.000 | 0.000 | 0.001 | 0.001 | 0.000 |  | |
| *Firmicutes* | *Bacilli* | *Enterococcus* | 0.005 | 0.003 | 0.000 | 0.000 | 0.000 | 0.000 | 0.000 | 0.000 | 0.001 | 0.000 | 0.000 |  | |
| *Firmicutes* | *Bacilli* | *Lactobacillus* | 0.010 | 0.019 | 0.000 | 0.000 | 0.000 | 0.000 | 0.000 | 0.000 | 0.000 | 0.000 | 0.000 |  | |
| *Firmicutes* | *Bacilli* | *Listeria* | 0.004 | 0.001 | 0.000 | 0.000 | 0.000 | 0.000 | 0.000 | 0.000 | 0.000 | 0.000 | 0.000 |  | |
| *Firmicutes* | *Bacilli* | *Streptococcus* | 0.000 | 0.000 | 0.000 | 0.000 | 0.000 | 0.000 | 0.000 | 0.001 | 0.001 | 0.000 | 0.000 |  | |
| *Firmicutes* | *Bacilli* | *Paenibacillus* | **3.219** | **4.304** | **1.671** | **1.636** | **0.147** | **0.313** | **0.290** | **0.614** | **6.736** | **7.219** | **0.264** |  | |
| *Firmicutes* | *Bacilli* | *Saccharibacillus* | 0.000 | 0.000 | 0.003 | 0.000 | 0.000 | 0.084 | 0.000 | 0.000 | 0.000 | 0.000 | 0.000 |  | |
| *Firmicutes* | *Bacilli* | *Staphylococcus* | 0.008 | 0.013 | 0.000 | 0.008 | 0.002 | 0.021 | 0.004 | 0.000 | 0.000 | 0.004 | 0.000 |  | |
| *Myxococcota* | *Polyangia* | *Pajaroellobacter* | 0.004 | 0.000 | 0.000 | 0.000 | 0.000 | 0.000 | 0.000 | 0.000 | 0.000 | 0.000 | 0.000 |  | |
| *Proteobacteria* | *Alphaproteobacteria* | *Unidentified_Acetobacteraceae* | 0.000 | 0.000 | 0.000 | 0.000 | 0.000 | 0.000 | 0.000 | 0.000 | 0.003 | 0.000 | 0.000 |  | |
| *Proteobacteria* | *Alphaproteobacteria* | *Roseomonas* | 0.083 | 0.000 | 0.000 | 0.000 | **0.104** | 0.010 | 0.001 | 0.019 | 0.006 | 0.000 | 0.038 |  | |
| *Proteobacteria* | *Alphaproteobacteria* | *Brevundimonas* | 0.000 | 0.000 | 0.001 | 0.002 | 0.000 | 0.000 | 0.000 | 0.000 | 0.000 | 0.000 | 0.000 |  | |
| *Proteobacteria* | *Alphaproteobacteria* | *Caulobacter* | 0.000 | 0.000 | 0.000 | 0.000 | 0.000 | 0.000 | 0.000 | 0.000 | 0.000 | 0.001 | 0.000 |  | |
| *Proteobacteria* | *Alphaproteobacteria* | *Uncultured_Caulobacteraceae* | 0.001 | 0.000 | 0.000 | 0.000 | 0.002 | 0.001 | 0.001 | 0.000 | 0.002 | 0.001 | 0.000 |  | |
| *Proteobacteria* | *Alphaproteobacteria* | *Methylobacterium* | **0.267** | 0.090 | **0.206** | 0.033 | 0.047 | 0.050 | 0.008 | **0.392** | **0.903** | **0.441** | **0.140** |  | |
| *Proteobacteria* | *Alphaproteobacteria* | *Rhizobium* | 0.001 | 0.069 | 0.008 | 0.001 | 0.000 | **0.797** | 0.025 | 0.000 | 0.079 | **0.130** | 0.000 |  | |
| *Proteobacteria* | *Alphaproteobacteria* | *Aureimonas* | 0.000 | 0.000 | 0.000 | 0.000 | 0.000 | 0.001 | 0.000 | 0.000 | 0.032 | 0.000 | 0.000 |  | |
| *Proteobacteria* | *Alphaproteobacteria* | *Bradyrhizobium* | 0.012 | 0.000 | 0.000 | 0.000 | 0.000 | 0.000 | 0.004 | 0.000 | 0.002 | 0.003 | 0.000 |  | |
| *Proteobacteria* | *Alphaproteobacteria* | *Unidentified_Sphingomonadaceae* | 0.078 | 0.000 | 0.006 | 0.005 | 0.000 | 0.001 | 0.000 | 0.014 | **0.175** | 0.007 | 0.026 |  | |
| *Proteobacteria* | *Alphaproteobacteria* | *Novosphingobium* | **1.204** | **1.642** | **2.564** | **0.836** | **1.691** | **0.624** | 0.030 | **0.460** | **0.176** | **6.171** | 0.001 |  | |
| *Proteobacteria* | *Alphaproteobacteria* | *Sphingomonas* | **2.469** | **0.719** | **2.197** | **0.183** | **1.115** | **1.751** | 0.039 | **2.679** | **20.672** | **3.920** | **1.071** |  | |
| *Proteobacteria* | *Gammaproteobacteria* | *Achromobacter* | 0.000 | 0.000 | 0.000 | 0.004 | 0.000 | 0.000 | 0.000 | 0.000 | 0.000 | 0.000 | 0.000 |  | |
| *Proteobacteria* | *Gammaproteobacteria* | *Castellaniella* | 0.000 | 0.004 | 0.000 | 0.001 | 0.000 | 0.000 | 0.001 | 0.000 | 0.000 | 0.004 | 0.000 |  | |
| *Proteobacteria* | *Gammaproteobacteria* | *Burkholderia* | **0.581** | 0.028 | 0.000 | 0.006 | 0.000 | 0.000 | 0.002 | 0.000 | 0.000 | 0.011 | 0.000 |  | |
| *Proteobacteria* | *Gammaproteobacteria* | *Ralstonia* | 0.059 | 0.000 | 0.001 | 0.010 | 0.000 | 0.001 | 0.021 | 0.000 | 0.000 | **0.282** | 0.000 |  | |
| *Proteobacteria* | *Gammaproteobacteria* | *Unidentified_Comamonadaceae* | 0.042 | **1.564** | 0.012 | 0.008 | 0.006 | 0.051 | 0.039 | 0.009 | 0.028 | 0.091 | 0.000 |  | |
| *Proteobacteria* | *Gammaproteobacteria* | *Curvibacter* | 0.003 | 0.000 | 0.002 | 0.002 | 0.000 | 0.000 | 0.002 | 0.001 | 0.000 | 0.000 | 0.000 |  | |
| *Proteobacteria* | *Gammaproteobacteria* | *Delftia* | 0.000 | 0.064 | 0.000 | 0.000 | 0.003 | 0.000 | 0.000 | 0.000 | 0.000 | 0.000 | 0.000 |  | |
| *Proteobacteria* | *Gammaproteobacteria* | *Tepidimonas* | 0.000 | 0.014 | 0.000 | 0.000 | 0.004 | 0.000 | 0.000 | 0.000 | 0.000 | 0.005 | 0.000 |  | |
| *Proteobacteria* | *Gammaproteobacteria* | *Uncultured_Neisseriaceae* | 0.000 | 0.000 | 0.000 | 0.000 | 0.000 | 0.000 | 0.000 | 0.000 | 0.000 | 0.001 | 0.000 |  | |
| *Proteobacteria* | *Gammaproteobacteria* | *Massilia* | **1.622** | 0.006 | 0.024 | 0.067 | 0.061 | **0.335** | **1.367** | **0.141** | **4.856** | **4.094** | **0.183** |  | |
| *Proteobacteria* | *Gammaproteobacteria* | *Unidentified_Enterobacterales* | 0.001 | 0.001 | 0.000 | 0.000 | 0.000 | 0.000 | 0.000 | 0.000 | 0.000 | 0.000 | 0.000 |  | |
| *Proteobacteria* | *Gammaproteobacteria* | *Unidentified_Enterobacteriaceae* | 0.009 | 0.004 | 0.000 | 0.000 | 0.000 | 0.000 | 0.000 | 0.000 | 0.000 | 0.002 | 0.000 |  | |
| *Proteobacteria* | *Gammaproteobacteria* | *Escherichia-Shigella* | 0.013 | 0.033 | 0.008 | 0.015 | 0.004 | 0.033 | 0.007 | 0.010 | 0.039 | 0.016 | 0.000 |  | |
| *Proteobacteria* | *Gammaproteobacteria* | *Unidentified_Erwiniaceae* | 0.000 | 0.032 | 0.035 | 0.013 | **1.329** | 0.031 | 0.003 | 0.001 | 0.000 | 0.002 | 0.000 |  | |
| *Proteobacteria* | *Gammaproteobacteria* | *Pantoea* | **33.929** | **73.568** | **72.498** | **90.470** | **85.880** | **67.302** | **88.982** | **91.233** | **1.871** | **62.389** | **80.656** |  | |
| *Proteobacteria* | *Gammaproteobacteria* | *Providencia* | 0.000 | 0.015 | 0.000 | 0.000 | 0.000 | 0.000 | 0.000 | 0.000 | 0.000 | 0.000 | 0.000 |  | |
| *Proteobacteria* | *Gammaproteobacteria* | *Unidentified_Yersiniaceae* | **0.128** | **0.136** | 0.010 | 0.064 | 0.000 | 0.001 | 0.060 | 0.000 | 0.022 | 0.081 | 0.000 |  | |
| *Proteobacteria* | *Gammaproteobacteria* | *Acidibacter* | 0.001 | 0.000 | 0.000 | 0.000 | 0.000 | 0.000 | 0.000 | 0.000 | 0.000 | 0.000 | 0.000 |  | |
| *Proteobacteria* | *Gammaproteobacteria* | *Halomonas* | 0.000 | 0.000 | 0.001 | 0.000 | 0.000 | 0.000 | 0.000 | 0.000 | 0.001 | 0.000 | 0.000 |  | |
| *Proteobacteria* | *Gammaproteobacteria* | *Acinetobacter* | 0.001 | 0.000 | 0.001 | 0.000 | 0.000 | 0.003 | 0.000 | 0.000 | 0.004 | 0.002 | 0.000 |  | |
| *Proteobacteria* | *Gammaproteobacteria* | *Enhydrobacter* | 0.000 | 0.000 | 0.000 | 0.000 | 0.000 | 0.000 | 0.000 | 0.000 | 0.006 | 0.001 | 0.000 |  | |
| *Proteobacteria* | *Gammaproteobacteria* | *Pseudomonas* | **51.117** | **0.938** | **11.268** | **0.351** | **5.213** | **25.681** | **6.480** | **0.312** | **13.276** | **0.809** | **12.376** |  | |
| *Proteobacteria* | *Gammaproteobacteria* | *Chujaibacter* | 0.000 | 0.000 | 0.000 | 0.000 | 0.000 | 0.000 | 0.001 | 0.000 | 0.000 | 0.004 | 0.000 |  | |
| *Proteobacteria* | *Gammaproteobacteria* | *Rhodanobacter* | 0.000 | 0.000 | 0.000 | 0.001 | 0.000 | 0.000 | 0.003 | 0.000 | 0.000 | 0.000 | 0.000 |  | |
| *Proteobacteria* | *Gammaproteobacteria* | *Stenotrophomonas* | 0.005 | 0.007 | 0.000 | 0.000 | 0.000 | 0.000 | 0.004 | 0.000 | 0.000 | 0.001 | 0.000 |  | |
| *Proteobacteria* | *Gammaproteobacteria* | *Xanthomonas* | 0.024 | 0.000 | 0.000 | 0.000 | 0.000 | 0.000 | 0.001 | 0.000 | 0.000 | 0.000 | 0.000 |  | |

**Table S2.** Relative abundance of the bacterial genera isolated from G. max seed samples belonged to the undried seed, dried seed (0 month) and seed stored for 3-, 6- and 14 months at -20°C, 4°C and RT. Taxa occurring at >0.1% are highlighted in bold.

| **Taxonomic Level** | | | **Relative abundance (%)** | | | | | | | | | | |  |  |  |
| --- | --- | --- | --- | --- | --- | --- | --- | --- | --- | --- | --- | --- | --- | --- | --- | --- |
|  |  |  |  |  |  |  |  |  |  |  |  |  |  |  | |  |
| **Phylum** | **Class** | **Genus** | **Undried seed** | **0 month** | **3 months_-20°C** | **6 months_-20°C** | **14 months_-20°C** | **3 months_4°C** | **6 months_4°C** | **14 months_4°C** | **3 months_RT** | **6 months_RT** | **14 months_RT** | |  | |
|  |  |  |  |  |  |  |  |  |  |  |  |  |  |  |  | |
| *Unassigned_Bacteria* |  |  | 0.016 | 0.000 | 0.008 | 0.014 | 0.002 | 0.006 | 0.000 | 0.000 | 0.000 | 0.008 | 0.000 | |  | |
| *Actinobacteriota* | *Actinobacteria* | *Unidentified_Microbacteriaceae* | 0.000 | 0.000 | 0.000 | 0.000 | 0.000 | 0.000 | 0.026 | 0.000 | 0.000 | 0.000 | 0.000 | |  | |
| *Actinobacteriota* | *Actinobacteria* | *Curtobacterium* | **1.992** | **1.236** | **2.204** | **1.275** | **2.710** | **0.311** | **0.704** | **0.968** | **30.445** | **0.371** | **0.325** | |  | |
| *Actinobacteriota* | *Actinobacteria* | *Microbacterium* | **0.407** | 0.065 | **0.101** | **0.194** | **0.127** | 0.002 | 0.038 | **0.423** | **0.603** | **0.797** | 0.000 | |  | |
| *Actinobacteriota* | *Actinobacteria* | *Unidentified_Micrococcaceae* | 0.028 | 0.000 | 0.000 | 0.000 | 0.000 | 0.000 | 0.000 | 0.016 | 0.000 | 0.050 | 0.000 | |  | |
| *Bacteroidota* | *Bacteroidia* | *Siphonobacter* | 0.002 | 0.000 | 0.008 | **0.125** | 0.000 | **1.627** | 0.002 | 0.034 | **4.814** | 0.000 | 0.000 | |  | |
| *Bacteroidota* | *Bacteroidia* | *Spirosoma* | 0.000 | 0.000 | 0.000 | 0.000 | 0.022 | 0.000 | 0.000 | 0.000 | 0.000 | 0.000 | 0.000 | |  | |
| *Bacteroidota* | *Bacteroidia* | *Uncultured_Spirosomaceae* | 0.000 | 0.000 | 0.000 | 0.000 | 0.000 | 0.000 | 0.000 | 0.000 | 0.004 | 0.000 | 0.000 | |  | |
| *Bacteroidota* | *Bacteroidia* | *Chryseobacterium* | 0.000 | 0.000 | 0.000 | 0.000 | 0.000 | 0.000 | **2.946** | 0.000 | 0.000 | 0.010 | 0.000 | |  | |
| *Bacteroidota* | *Bacteroidia* | *Mucilaginibacter* | 0.012 | **0.246** | **0.841** | 0.002 | **0.149** | **0.115** | 0.002 | 0.000 | **2.962** | 0.020 | 0.000 | |  | |
| *Firmicutes* | *Bacilli* | *Bacillus* | 0.010 | **1.394** | **0.879** | **0.899** | **0.524** | **0.351** | **0.714** | **1.311** | **17.851** | **0.823** | **2.448** | |  | |
| *Firmicutes* | *Bacilli* | *Paenibacillus* | **1.688** | **1.085** | **2.293** | **1.988** | **3.499** | **0.184** | **5.384** | **11.201** | **0.208** | **3.174** | **3.634** | |  | |
| *Firmicutes* | *Bacilli* | *Saccharibacillus* | 0.000 | 0.000 | **0.335** | 0.000 | 0.000 | **0.647** | 0.000 | 0.000 | 0.000 | 0.000 | 0.000 | |  | |
| *Proteobacteria* | *Alphaproteobacteria* | *Roseomonas* | 0.012 | 0.000 | 0.000 | 0.000 | **1.533** | 0.083 | 0.000 | 0.063 | 0.000 | 0.000 | 0.000 | |  | |
| *Proteobacteria* | *Alphaproteobacteria* | *Methylobacterium* | 0.000 | 0.000 | 0.000 | 0.000 | 0.000 | 0.000 | 0.000 | 0.071 | 0.060 | 0.000 | 0.000 | |  | |
| *Proteobacteria* | *Alphaproteobacteria* | *Rhizobium* | 0.012 | 0.091 | 0.020 | 0.004 | 0.000 | **6.457** | 0.002 | 0.000 | **26.128** | **0.341** | 0.000 | |  | |
| *Proteobacteria* | *Alphaproteobacteria* | *Aureimonas* | 0.000 | 0.000 | 0.000 | 0.000 | 0.000 | 0.000 | 0.000 | **0.355** | **0.186** | 0.000 | 0.000 | |  | |
| *Proteobacteria* | *Alphaproteobacteria* | *Unidentified_Sphingomonadaceae* | 0.048 | 0.016 | 0.014 | 0.000 | 0.022 | 0.000 | 0.000 | 0.000 | 0.018 | 0.000 | 0.000 | |  | |
| *Proteobacteria* | *Alphaproteobacteria* | *Novosphingobium* | **3.467** | **6.919** | **6.978** | **6.121** | **4.985** | **1.079** | 0.077 | **0.930** | **9.275** | **3.473** | 0.000 | |  | |
| *Proteobacteria* | *Alphaproteobacteria* | *Sphingomonas* | **2.335** | **0.403** | **2.458** | **3.277** | **6.560** | 0.004 | **0.204** | **0.141** | **6.746** | **0.375** | **2.464** | |  | |
| *Proteobacteria* | *Gammaproteobacteria* | *Advenella* | 0.000 | 0.093 | 0.000 | 0.000 | 0.000 | 0.000 | 0.000 | 0.000 | 0.000 | 0.000 | 0.000 | |  | |
| *Proteobacteria* | *Gammaproteobacteria* | *Unidentified_Comamonadaceae* | 0.000 | 0.000 | 0.000 | 0.000 | 0.000 | **0.175** | 0.000 | 0.000 | 0.000 | 0.000 | 0.000 | |  | |
| *Proteobacteria* | *Gammaproteobacteria* | *Massilia* | **0.198** | 0.058 | 0.000 | 0.000 | 0.000 | 0.004 | **0.849** | 0.000 | 0.004 | **1.744** | **1.470** | |  | |
| *Proteobacteria* | *Gammaproteobacteria* | *Unidentified_Gammaproteobacteria* | 0.000 | 0.000 | 0.000 | 0.000 | 0.000 | 0.000 | 0.000 | 0.000 | 0.000 | 0.002 | 0.000 | |  | |
| *Proteobacteria* | *Gammaproteobacteria* | *Unidentified_Erwiniaceae* | **0.101** | 0.002 | 0.030 | 0.085 | **2.382** | 0.006 | 0.048 | 0.000 | 0.000 | 0.030 | 0.000 | |  | |
| *Proteobacteria* | *Gammaproteobacteria* | *Pantoea* | **86.995** | **73.090** | **80.739** | **85.391** | **69.181** | **48.228** | **86.938** | **84.488** | **0.192** | **87.279** | **86.424** | |  | |
| *Proteobacteria* | *Gammaproteobacteria* | *Pseudomonas* | **2.676** | **15.302** | **3.092** | **0.625** | **8.303** | **40.720** | **2.065** | 0.000 | **0.504** | **1.502** | **3.235** | |  | |

**Table S3.** P-values of the comparison between seed samples belonging to undried seed, dried seed (0 month) and seed stored for 3-, 6-, 14 months at -20°C, 4°C and RT. The significant differences were determined using pairwise-ANOSIM test for Alpha diversity (Observed features) and Beta diversity (Jaccard distance) metrics.

| **Group 1** | **Group 2** | **Alpha diversity (Observed features)** | **Beta diversity (Jaccard distance)** |  |
| --- | --- | --- | --- | --- |
|  |  |  |  |  |
| Undried seed | 3 months_-20°C | 0.0008 | 0.0010 |  |
|  | 3 months_4°C | 0.0051 | 0.0010 |  |
|  | 3 months_RT | 0.4897 | 0.0010 |  |
|  | 6 months_-20°C | 0.0001 | 0.0010 |  |
|  | 6 months_4°C | 0.0000 | 0.0010 |  |
|  | 6 months_RT | 0.0094 | 0.0010 |  |
|  | 14 months_-20°C | 0.0000 | 0.0010 |  |
|  | 14 months_4°C | 0.0001 | 0.0010 |  |
|  | 14 months_RT | 0.0001 | 0.0010 |  |
| Dried seed (0 month) | Undried seed | 0.3449 | 0.0010 |  |
|  | 3 months_-20°C | 0.0119 | 0.0010 |  |
|  | 3 months_4°C | 0.0555 | 0.0010 |  |
|  | 3 months_RT | 0.1195 | 0.0010 |  |
|  | 6 months_-20°C | 0.0004 | 0.0010 |  |
|  | 6 months_4°C | 0.0001 | 0.0010 |  |
|  | 6 months_RT | 0.1275 | 0.0010 |  |
|  | 14 months_-20°C | 0.0001 | 0.0010 |  |
|  | 14 months_4°C | 0.0002 | 0.0010 |  |
|  | 14 months_RT | 0.0001 | 0.0010 |  |
| 3 months_-20°C | 3 months_RT | 0.3053 | 0.0010 |  |
|  | 6 months_-20°C | 0.0000 | 0.0010 |  |
|  | 6 months_4°C | 0.0496 | 0.0010 |  |
|  | 6 months_RT | 0.0034 | 0.0010 |  |
|  | 14 months_-20°C | 0.1341 | 0.0010 |  |
|  | 14 months_4°C | 0.0014 | 0.0010 |  |
|  | 14 months_RT | 0.0109 | 0.0010 |  |
| 3 months_4°C | 3 months_-20°C | 0.0001 | 0.0050 |  |
|  | 3 months_RT | 0.0002 | 0.0010 |  |
|  | 6 months_-20°C | 0.0090 | 0.0010 |  |
|  | 6 months_4°C | 0.0004 | 0.0010 |  |
|  | 6 months_RT | 0.6429 | 0.0010 |  |
|  | 14 months_-20°C | 0.0002 | 0.0010 |  |
|  | 14 months_4°C | 0.0011 | 0.0010 |  |
|  | 14 months_RT | 0.0000 | 0.0010 |  |
| 3 months_RT | 6 months_-20°C | 0.0000 | 0.0010 |  |
|  | 6 months_4°C | 0.0000 | 0.0010 |  |
|  | 6 months_RT | 0.0004 | 0.0010 |  |
|  | 14 months_-20°C | 0.0000 | 0.0010 |  |
|  | 14 months_4°C | 0.0000 | 0.0010 |  |
|  | 14 months_RT | 0.0000 | 0.0010 |  |
| 6 months_-20°C | 6 months_RT | 0.1686 | 0.0010 |  |
|  | 14 months_-20°C | 0.0011 | 0.0010 |  |
|  | 14 months_4°C | 0.0954 | 0.0010 |  |
|  | 14 months_RT | 0.2076 | 0.0010 |  |
| 6 months_4°C | 6 months_-20°C | 0.0028 | 0.0010 |  |
|  | 6 months_RT | 0.0001 | 0.0010 |  |
|  | 14 months_-20°C | 0.7407 | 0.0010 |  |
|  | 14 months_4°C | 0.8620 | 0.0010 |  |
|  | 14 months_RT | 0.0946 | 0.0010 |  |
| 6 months_RT | 14 months_-20°C | 0.0001 | 0.0010 |  |
|  | 14 months_4°C | 0.0004 | 0.0010 |  |
|  | 14 months_RT | 0.0000 | 0.0010 |  |
| 14 months_-20°C | 14 months_RT | 0.5290 | 0.0010 |  |
| 14 months_4°C | 14 months_-20°C | 0.1341 | 0.0010 |  |
|  | 14 months_RT | 0.0157 | 0.0010 |  |

**Table S4.** Relative abundance of Pantoea and Pseudomonas isolated from undried and dried (0 month) G. max seed per replicate plate.

| **Index** | **Undried seed_Rep 1** | **Undried seed_Rep 2** | **Undried seed_Rep 3** | **0 month_Rep 1** | **0 month_Rep 2** | **0 month_Rep 3** |
| --- | --- | --- | --- | --- | --- | --- |
| *Pantoea* | 88.38405227 | 81.85613165 | 90.74354165 | 47.03248835 | 82.5337286 | 89.70294634 |
| *Pseudomonas* | 3.587633856 | 4.440680017 | 0 | 45.76199407 | 0.114949483 | 0.030249864 |

**Table S5.** P-values of the comparison of bacterial abundance between undried seed and dried seed (0 month) in planta and on plates. The Significant differences (p ≤ 0.05) were determined by one way ANOVA test and are highlighted in bold.

| **Genera** | **Undried seed vs Dried seed (0 month)** | |
| --- | --- | --- |
|  | ***In planta*** | **On plates** |
|  | **p-value** | |
| *Pseudomonas* | **2.96E-04** | 0.45534 |
| *Pantoea* | **0.00628** | 0.35985 |
| *Paenibacillus* | 0.6118 | 0.61534 |
| *Sphingomonas* | **0.03437** | 0.30515 |
| *Bacillus* | 0.16927 | 0.23303 |
| *Massilia* | **0.00366** | 0.36275 |
| *Novosphingobium* | 0.74169 | 0.3342 |
| *Curtobacterium* | **0.01514** | 0.48804 |
| *Mucilaginibacter* | 0.5555 | 0.13057 |
| *Burkholderia* | 0.28791 | Not detected on plates |
| *Methylobacterium* | **0.02933** | Not detected on plates |
| *Spirosoma* | 0.48268 | Not detected on plates |
| *Siphonobacter* | 0.54442 | 0.3739 |
| *Hymenobacter* | 0.97195 | Not detected on plates |
| *Microbacterium* | 0.39473 | 0.29067 |
| *Unidentified_Yersiniaceae* | 0.92192 | Not detected on plates |
| *Roseomonas* | 0.1481 | 0.26057 |
| *Unidentified_Sphingomonadaceae* | 0.09836 | 0.27838 |
| *Ralstonia* | 0.29174 | Not detected on plates |
| *Unidentified_Microbacteriaceae* | 0.38808 | Not detected on plates |
| *Unidentified_Comamonadaceae* | 0.3117 | Not detected on plates |
| *Rhizobium* | 0.23254 | 0.19177 |
| *Unidentified_Erwiniaceae* | **0.03651** | **2.87E-04** |
| *Unidentified_Micrococcaceae* | 0.91334 | 0.3739 |

# **Supplementary figures**


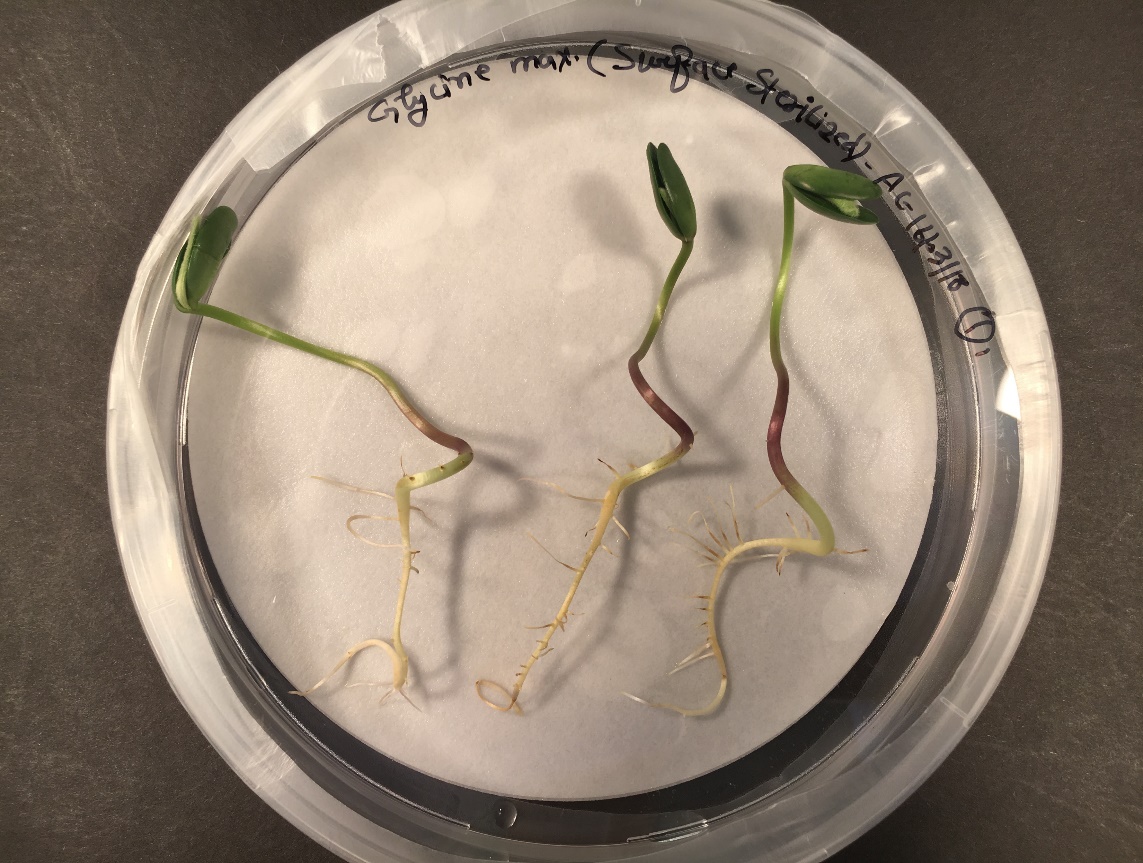


**Figure S1.** *G. max* seedlings at the unfolded cotyledon growth stage.


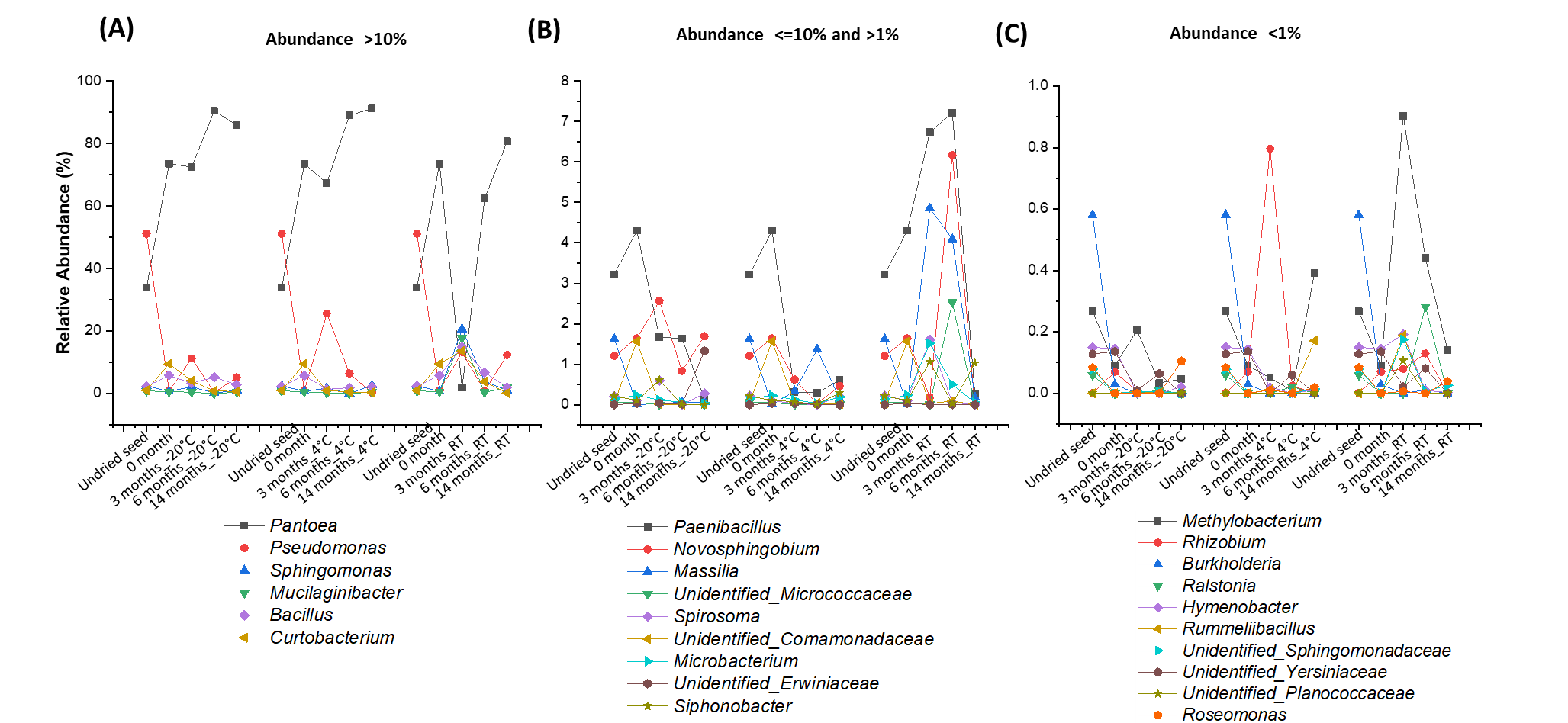


**Figure S2.** Line graphs showing the changes in abundance of the bacterial genera that were present A) with >10% relative abundance B) with <1% relative abundance C) <=10% and >1% relative abundance in undried seed, dried seed (0 month) and seed stored for 3-, 6-, and 14 months at -20°C, 4°C and RT in planta. The abundance was estimated based on the most abundant bacteria genera across all time points.


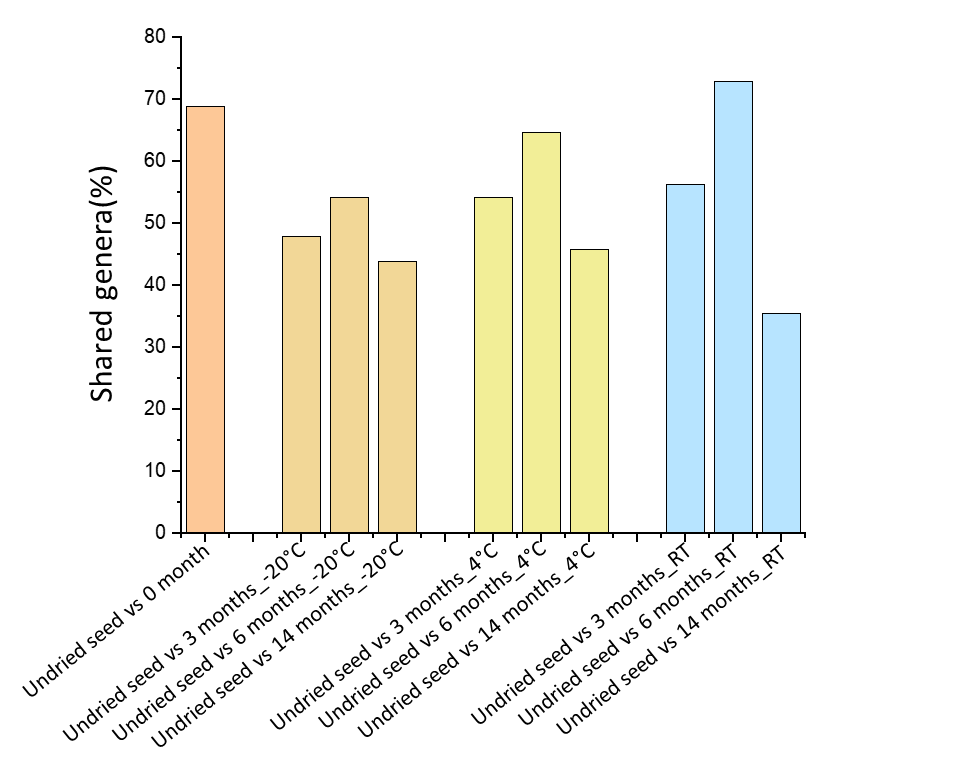


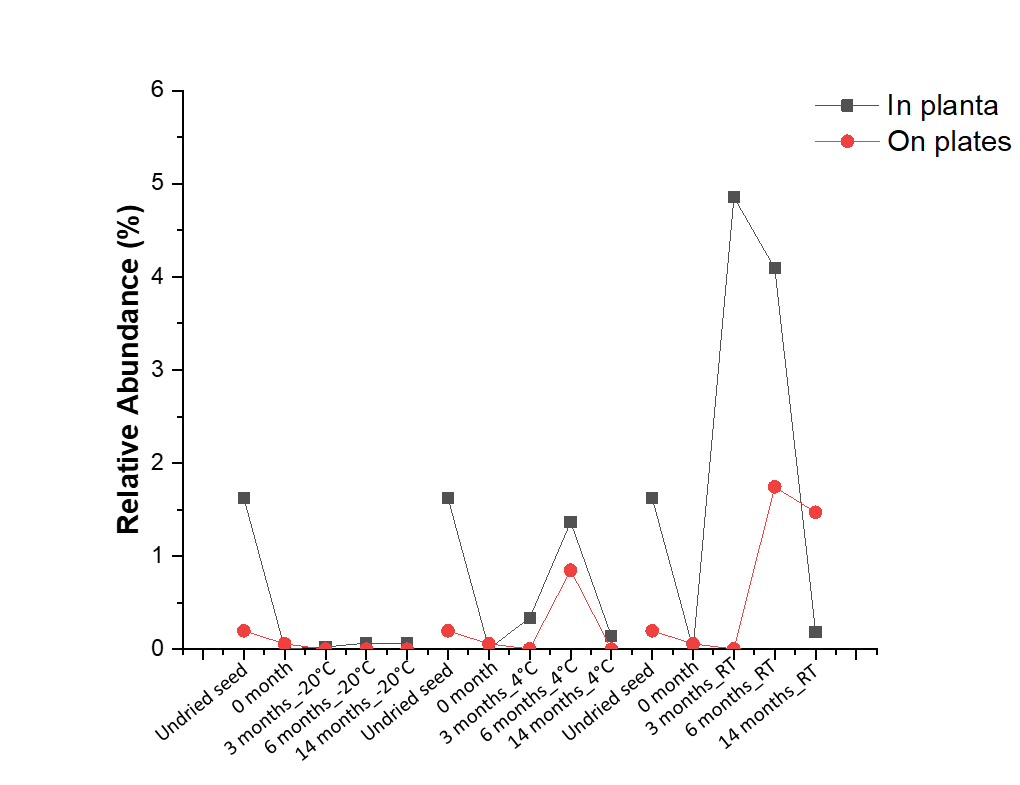


**Figure S3.** Percentage of genera shared between undried seed, dried seed (0 month) and seed stored for 3-, 6-, and 14 months at -20°C, 4°C and RT.

**Figure S4.** Relative abundance of Massilia in undried seed, dried seed (0 month) and seed stored for 3-,6-, and 14 months at -20°C, 4°C and RT.
